# Crol contributes to PRE-mediated repression and Polycomb group proteins recruitment in *Drosophila*

**DOI:** 10.1093/nar/gkad336

**Published:** 2023-05-04

**Authors:** Maksim Erokhin, J Lesley Brown, Dmitry Lomaev, Nadezhda E Vorobyeva, Liangliang Zhang, Lika V Fab, Marina Yu Mazina, Ivan V Kulakovskiy, Rustam H Ziganshin, Paul Schedl, Pavel Georgiev, Ming-an Sun, Judith A Kassis, Darya Chetverina

**Affiliations:** Group of Chromatin Biology, Institute of Gene Biology, Russian Academy of Sciences, 34/5 Vavilov Street, Moscow 119334, Russia; Eunice Kennedy Shriver National Institute of Child Health and Human Development, National Institutes of Health, Bethesda, MD 20892, USA; Group of Epigenetics, Institute of Gene Biology, Russian Academy of Sciences, 34/5 Vavilov Street, Moscow 119334, Russia; Group of transcriptional complexes dynamics, Institute of Gene Biology, Russian Academy of Sciences, Moscow, Russia; Institute of Comparative Medicine, College of Veterinary Medicine, Yangzhou University, Yangzhou, Jiangsu, China; Group of Chromatin Biology, Institute of Gene Biology, Russian Academy of Sciences, 34/5 Vavilov Street, Moscow 119334, Russia; Group of hormone-dependent transcription regulation, Institute of Gene Biology, Russian Academy of Sciences, Moscow, Russia; Vavilov Institute of General Genetics, Russian Academy of Sciences, Moscow119991, Russia; Shemyakin-Ovchinnikov Institute of Bioorganic Chemistry, Russian Academy of Sciences, Moscow 117997, Russia; Department of Molecular Biology Princeton University, Princeton, NJ 08544, USA; Department of Control of Genetic Processes, Institute of Gene Biology, Russian Academy of Sciences, 34/5 Vavilov Street, Moscow 119334, Russia; Institute of Comparative Medicine, College of Veterinary Medicine, Yangzhou University, Yangzhou, Jiangsu, China; Eunice Kennedy Shriver National Institute of Child Health and Human Development, National Institutes of Health, Bethesda, MD 20892, USA; Group of Epigenetics, Institute of Gene Biology, Russian Academy of Sciences, 34/5 Vavilov Street, Moscow 119334, Russia

## Abstract

The Polycomb group (PcG) proteins are fundamental epigenetic regulators that control the repressive state of target genes in multicellular organisms. One of the open questions is defining the mechanisms of PcG recruitment to chromatin. In *Drosophila*, the crucial role in PcG recruitment is thought to belong to DNA-binding proteins associated with Polycomb response elements (PREs). However, current data suggests that not all PRE-binding factors have been identified. Here, we report the identification of the transcription factor Crooked legs (Crol) as a novel PcG recruiter. Crol is a C2H2-type Zinc Finger protein that directly binds to poly(G)-rich DNA sequences. Mutation of Crol binding sites as well as *crol* CRISPR/Cas9 knockout diminish the repressive activity of PREs in transgenes. Like other PRE-DNA binding proteins, Crol co-localizes with PcG proteins inside and outside of H3K27me3 domains. Crol knockout impairs the recruitment of the PRC1 subunit Polyhomeotic and the PRE-binding protein Combgap at a subset of sites. The decreased binding of PcG proteins is accompanied by dysregulated transcription of target genes. Overall, our study identified Crol as a new important player in PcG recruitment and epigenetic regulation.

## INTRODUCTION

Epigenetic control is required to establish and maintain correct patterns of gene expression that determine cellular identity and prevent the development of pathologies in multicellular organisms (1). Polycomb group (PcG) proteins are transcriptional repressors that control chromatin state of facultative heterochromatin (2–5). PcG genes were first identified genetically in *Drosophila* through studies on the Hox genes that specify segment identity during development (3,5–7). Mutations in PcG encoding genes led to homeotic transformations due to derepression of the Hox genes (3,5–7). After these initial discoveries, it was found that PcG proteins control transcription of many developmental genes, and dysfunction of PcG genes is observed in many diseases, including cancer (8–13).

Most of the known PcG proteins are organized into multiprotein complexes that interact with chromatin and mediate repression of gene transcription (4,14). Polycomb repressive complex 1 (PRC1) has four main subunits: Polycomb (Pc), Polyhomeotic (Ph), Sex combs extra (Sce, also known as dRing), and Posterior sex combs (Psc) (15–17). PRC2 contains Enhancer of zeste (E(z)), Suppressor of zeste 12 (Su(z)12), Extra sex combs (Esc) and Chromatin assembly factor 1 (Caf1) (18,19). The E(z) subunit is a histone methyltransferase that catalyzes the H3K27me3 modification (18,19) specific for chromatin regions repressed by the PcG (20,21).

PRC1 and PRC2 complexes do not contain DNA-binding subunits, and the mechanism of recruitment of PcG complexes to chromatin is an active area of research. In *Drosophila*, PcG proteins are recruited to DNA via specialized regulatory elements known as Polycomb response elements (PREs) a.k.a. silencers (6,22–24). Current evidence indicates that core subunits of the PcG complexes are also present in active chromatin: promoters and potential enhancers, where they have been reported to play both an activating and repressive role (25–31).

In *Drosophila*, the core sequence of a PRE consists of binding sites for various DNA-binding proteins that mediate the recruitment of PcG proteins in a combinatorial and redundant fashion (22–24). The PRE-binding protein Pleiohomeotic (Pho) (32,33) is present in a complex with Sfmbt (PhoRC) (34–36) and plays a key role in PRC1 and PRC2 recruitment to a subset of PREs (29,37,38). However, Pho-binding sites alone are not sufficient for PcG recruitment and additional DNA-binding factors are required for PRE function (22,23). Other PRE-binding proteins identified include Combgap (Cg) (39), Pipsqueak (Psq) (40,41), Pleiohomeotic-like (Phol) (42), GAGA-factor (GAF) (43–45), Sp1 factor for pairing-sensitive silencing (Spps) (46), Zeste (Z) (16,47), Grainyhead (Grh) (48), Dorsal switch protein 1 (Dsp1) (49), Alcohol dehydrogenase transcription factor 1 (Adf1) (50).

Despite the discovery of the aforementioned PRE-binding factors, evidence suggests that additional currently unidentified DNA-binding proteins are involved in the recruitment of PcG proteins (51,52). In this study, we identify the polydactyl-zinc finger protein Crol as a new PRE-DNA binding factor. We show that Crol interacts directly with Cg and co-purifies with Pho (PhoRC) and Ph (PRC1) subunits. Genome-wide studies indicate that Crol is required for Ph and Cg recruitment at a subset of sites, while a transgene assay directly demonstrates the participation of Crol in PRE-mediated repressive activity.

## MATERIALS AND METHODS

### Yeast two-hybrid screening (Y2H)

The Cg (CG8367) PF 31–467 aa isoform was cloned into pGBT9 vector (Clontech) to make a fusion with the GAL4 DNA-binding domain. The cDNA fragments encoding the C2H2-type zinc finger (C2H2-ZFP) candidate proteins were cloned into pGAD24 vector (Clontech) to combine with the GAL4 activating domain. The full-length cDNA was used for Crol (CG14938, isoform A). Other proteins tested are listed in Supplementary File 1.

The Y2H assay was performed as previously described (53). Briefly, for the growth assays, plasmids were transformed into pJ69-4A strain by the lithium acetate method following the standard Clontech protocol and plated on the nonselective media lacking tryptophan and leucine. After 3 days of growth at 30°C, plates were replicated on selective media: 1 – lacking tryptophan, leucine, and histidine in the presence of 5 mM 3-aminotriazole (−3 + 3AT); 2 – lacking tryptophan, leucine, histidine, and adenine (-4). Each assay was repeated three times and growth was compared after 2, 4 and 7 days. Based on the extent of growth the interactions are scored as strong (detected on day 2, ‘+++’), intermediate (detected on day 4, ‘++’) or weak (detected on day 7, ‘+’). ‘−’ indicates no growth.

### Antibodies

Antibodies against the following proteins were raised in rabbits: CrolN (a.a. 1–188, isoform PA), CrolC (a.a. 726–962, isoform PA), E(z) (a.a. 8–184, isoform PA). Antigens for antibody production were expressed as 6× His-tagged fusion proteins in *Escherichia coli*, affinity purified on Ni Sepharose 6 Fast Flow (GE Healthcare), according to the manufacturer's protocol, and injected into rabbits following standard immunization procedures. Antibodies were affinity-purified from serum on the same antigen as was used for the immunization and tested by immunoprecipitation/Western blotting (IP/WB) to confirm their specificity (Supplementary File 2). IP/WBs were prepared as described previously (54). Proteins were detected using the ECL Plus Western Blotting substrate (Pierce).

Other antibodies used in this study were created previously, with details provided in Supplementary File 3.

### ChIP-qPCR

Chromatin immunoprecipitation (X-ChIP) was prepared as described previously (55). For each experiment, 150–200 mg of third instar larvae or 0–16 h embryos were collected. The material was homogenized and crosslinked with 1.8% formaldehyde for 15 min. Crosslinking was quenched with glycine and homogenate was cleared by passing through 100-μm nylon cell strainer (BD Falcon), washed, and sonicated to generate 200–600 bp DNA fragments. Chromatin was preincubated with A-Sepharose. One aliquot (1/10 volume) of chromatin extract after preincubation with Sepharose was kept as a control sample (Input). The resulting chromatin was used for 6–8 individual incubations with antibodies. Chromatin was incubated with specific antibodies-Sepharose at 4°C overnight. A control incubation was made with IgG of nonimmunized animal (rabbit). All ChIP experiments were made in biological triplicate. After washing and eluting from beads, the crosslinking was reversed and the DNA was isolated.

The enrichment of specific DNA fragments was analyzed by real-time qPCR, using a C1000™ Thermal Cycler with CFX96 real-time PCR detection module (Bio-Rad) or a StepOne Plus Thermal Cycler (Applied Biosystems, United States). Detailed protocol for ChIP-qPCR is described in Supplementary File 4. Primers used in ChIP/real-time PCR analyses are listed in Supplementary File 5.

### ChIP-seq

Brains and imaginal discs from third instar larvae (10 larvae per sample) were crosslinked with 2% formaldehyde for 15 min. Crosslinking was quenched with glycine. The samples were homogenized and sonicated to generate 200–500 bp DNA fragments. A percentage of the sample was removed as the input control. Chromatin was pre-cleared with protein A sepharose beads and then incubated overnight with antibody at 4°C. Chromatin was then precipitated with Protein A Sepharose beads for 1 h at 4°C. After washing and eluting from beads the crosslinking was reversed and DNA was isolated.

ChIP-seq libraries were obtained using the NEBNext Ultra™ II DNA library preparation kit (New England Biolabs) or with the Thruplex DNA-seq and single index kits (Takara) following the manufacturer's instructions. Samples were sequenced by 50bp or 100bp single-end sequencing with HiSeq2500 (Illumina) or with NovaSeq6000 sequencer. Detailed procedure for ChIP-seq is described in Supplementary File 4.

Reads were trimmed using Trim Galore v0.6.5 and then aligned to the reference genome (dm3) using Bowtie v2.3.5 (56) with default parameters. The PCR duplicates for each dataset were removed using the *rmdup* function of samtools v1.13 (57). The data reproducibility between biological replicates was confirmed, then reads from replicates were pooled together for further analysis. Peak calling was performed with MACS v2.2.5 (58) with parameters: -g dm -q 0.05 –nomodel –extsize 300. H3K27me3 was called as broad peaks, while all other factors were called as narrow peaks. Differential binding analysis was performed using DiffBind2 (59), with summit set as 250. Overlap of peaks were performed using the window function from BEDTools v2.29.2 (60). ChIP-seq tracks were visualized using IGV (61), and heatmaps were created using DeepTools v3.5.1 (62).

### Motif analysis

To predict the recognized DNA motif of Crol from its zinc finger arrays, the web server (https://zf.princeton.edu) developed by Persikov *et al.* was used (63). To perform motif discovery from ChIP-seq peaks, we ordered the peaks by *q*-value and extracted sequences of 300 bp windows centered at the peak summits. MEME v5.4.1 (64) was executed with sequences from the top 500 peaks with default parameters, ChIPMunk (65) was executed with sequences from the top 500 and top 1000 peaks and provided with the peak summit (midpoint) location.

### RNA-seq

For the extraction of RNA, the third instar larvae of wild-type (Oregon) and *crol*-KO mutants were collected in a PBS buffer (40 larvae per sample), in three biological repeats. Total RNA was extracted with the TRI reagent (Ambion). PolyA comprising RNA fraction was isolated and prepared for sequencing with the NEBNext Ultra™ II Directional RNA Library Prep Kit. New generation sequencing was performed by Evrogen (evrogen.ru) with the Illumina NovaSeq6000 sequencer. For each RNA‐seq library approximately 20–25 millions of paired-end reads were obtained.

Reads were trimmed using Trim Galore v0.6.1, and then aligned to the reference genome (dm3) using STAR v2.7.3a (66). To identify differentially expressed genes, we first obtained the gene-level read counts with the *featureCount* function of subread v2.0.0 (67), then identified differentially expressed genes with FDR < 0.05 and |log_2_foldChange|>1 using DESeq2 v1.22.2 (68). Gene Ontology (GO) enrichment analysis was performed using metascape (69). The Transcripts Per Million (TPM) value for each gene was calculated using RSEM v1.3.2 (70). Representative RNA-Seq tracks were visualized using IGV (61).

### Electrophoretic mobility shift assay (EMSA)

Crol protein–DNA binding domain (zinc finger domains 7–16) was expressed as a fusion with MBP domain in pMAL-C5X (New England Biolabs). As a control empty pMAL-C5X vector expressing only MBP was used. *Escherichia coli* BL21 cells were transformed with plasmids expressing Crol-MBP or pMAL-C5X and were grown in LB media to an *A*_600_ of 1.0 at 37°C and then induced with 1 mM IPTG at 18°C overnight. ZnCl_2_ was added to final concentration 0.2 mM before induction. Proteins were purified using standard procedures. Briefly, the cells expressing recombinant proteins were centrifuged, resuspended in 1–2 ml of buffer A (20 mM HEPES–KOH pH7.5; 150 mM NaCl; 10mM MgCl_2_; 0.1 mM ZnCl_2_; 0.1% NP 40; 10% glycerol (w/v); 1 mM DTT, 1:25 Roche Protease Inhibitor Cocktail 11836145001) and disrupted by sonication. The lysate was cleared by centrifugation and incubated with Amylose Resin (New England Biolabs) in a starting buffer (20 mM HEPES–KOH pH7.5; 150 mM NaCl; 10mM MgCl_2_; 0.1 mM ZnCl_2_; 0.1% NP 40; 10% glycerol (w/v); 1 mM DTT, 1:25 Roche Protease Inhibitor Cocktail 11836145001). After washing three times with 500-NaCl buffer (20 mM HEPES–KOH pH7.5; 500 mM NaCl; 10mM MgCl_2_; 0.1 mM ZnCl_2_; 0.1% NP 40; 10% Glycerol (w/v); 1mM DTT, 1:25 Roche Protease Inhibitor Cocktail 11836145001), the bound proteins were eluted with maltose-containing buffer (20 mM HEPES–KOH pH7.5; 200 mM NaCl; 10mM MgCl_2_; 0.1 mM ZnCl_2_; 10 mM maltose; 0.5 mM DTT). After elution the Roche Protease Inhibitor Cocktail 11836145001 was added.

Aliquots of purified recombinant proteins (0.05–1.2 μg) were incubated with fluorescently labeled DNA fragments (60–80 ng) in the presence of nonspecific binding competitor—1 ng of poly(dI-dC). DNA fragments were labeled using PCR with primers containing the Cy5 fluorophore. Signals for Cy5 were detected at the Ex 630 nm/Em 700 nm. The sequences of 20xG, Control, 20 bp-evePRE, 20 bp-evePREmut containing fragments obtained by PCR are given in Supplementary File 4. Incubation was performed in 1× PBS (pH 8.0) containing 5 mM MgCl_2_, 0.1 mM ZnSO_4_, 1 mM DTT, 0.1% NP-40 and 10% glycerol at room temperature for 30 min. The mixtures were then resolved by nondenaturing 5% PAGE (79 AA:1 BAA) in 0.5× TBE buffer at 5 V/cm.

### Generation of control, eve-wt and eve-mut transgenic lines

The details of cloning of the Control, eve-wt and eve-mut plasmid constructs are described in Supplementary File 4. The constructs were injected into embryos of attP2 line (71). The resulting flies were crossed with yacw*^1118^* flies, and the transgenic progeny was identified by their eye pigmentation. For phenotype analysis of *white* gene expression level, we visually determined the degree of pigmentation in the eyes of 3- to 5-day-old males, with reference to standard color scales. Pigmentation of all flies was analyzed in homozygotes (P/P). All flies were maintained at 25°C on the standard yeast medium.

### Generation of *crol* knockout (*crol*-KO) flies by CRISPR-cas9–induced homologous recombination

Generation of CRISPR-crol-target plasmid coding gRNAs to *crol* gene and HR-crol plasmid for homologous recombination (Supplementary File 6, Figure S1) are described in Supplementary File 4. Plasmids mixture (10:1 - HR-*crol*: CRISPR-*crol*-target, total concentration 500 ng/μl) was injected into embryos of y[1] M(Act5C-Cas9.P.RFP-)ZH-2A w[1118] DNAlig4[169] line (Bloomington Drosophila Stock Center #58492). These flies express Cas9 under control of the Actin5C promoter. Injected embryos were grown to adulthood and crossed with *y^−^w^1118^*. Flies with potential *crol* deletions were identified by fluorescent DsRed expression using Leica MZ16F Stereomicroscope. The scheme of experiment with homologous recombination of the *crol* gene after co-injection of CRISPR-*crol*-target and HR-*crol* plasmids into Cas9-expressing flies is demonstrated on Supplementary File 6 (Figure S2). The obtained flies were balanced against a CyO, P(w[+mC] = Tb[1])Cpr[CyO-A] balancer (Bloomington stock center #56552). Successful deletions and correct homologous recombination were verified by qPCR using DNA extracted from transgenic flies (Supplementary File 6, Figure S2). All flies were maintained at 25°C on the standard yeast medium.

## RESULTS

### Crol interacts with Polycomb group proteins

**Figure 1.**
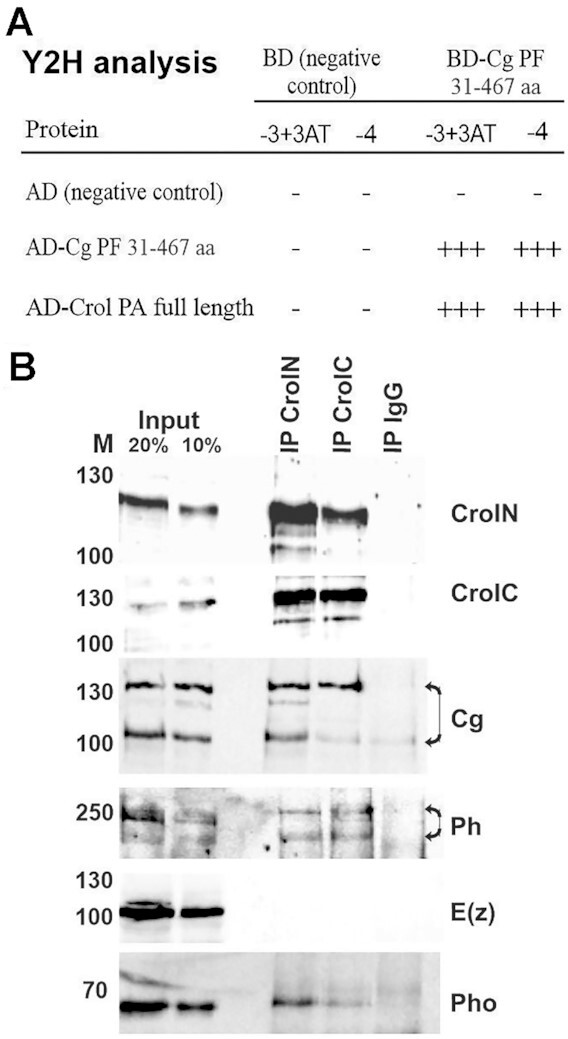
Crol interacts with PcG proteins. (**A**) Y2H-analysis of Cg-Crol interaction. Two types of selective media were used: lacking tryptophan, leucine, and histidine in the presence of 5 mM 3-aminotriazole (−3 + 3AT) and lacking tryptophan, leucine, histidine, and adenine (−4). The ‘+++’ indicates that strong growth was detected on second day; ‘−’—no growth detected on seventh day. The Cg-Cg interaction was demonstrated previously and served as positive control. (**B**) IP/WB analysis of interactions between Crol and Cg, Ph, E(z) and Pho. S2 *Drosophila* cell nuclear extracts were incubated with CrolN (IP CrolN), CrolC (IP CrolC) rabbit antibodies or IgG of a non-immunized rabbit (IP IgG). Lysates (Input 20% and 10%), precipitated fractions (IP CrolN, IP CrolC and IP IgC) are indicated above the blots. Antibodies used for Western blots are indicated on the right side from the blots.

To identify novel proteins that participate in recruitment of PcG proteins, we employed the Y2H assay using the PRE-binding protein Cg as a bait. Cg is tightly connected to PRC1 complex (39,72). Apart from PRC1 subunits, its interactome includes several known PRE-binding proteins as well as DNA-binding factors with uncharacterized connection to PcG system (72). As candidate DNA-binding proteins, we tested a number of C2H2-ZFPs previously identified in the Cg protein complex (72) (Supplementary File 1). Cg was fused to the DNA binding domain of yeast GAL4 protein, while each C2H2-ZFP was fused to the activator domain of GAL4. As a result, we identified Crol (Сrooked legs, CG14938) as a direct Cg interactor (Figure 1A). Interestingly, several studies support the potential role of Crol in Polycomb-dependent transcriptional repression. Crol was described as an ecdysone-induced repressor of *wg* transcription in wing imaginal discs (73). In addition, its knockdown was shown to disrupt the formation of Polycomb bodies (74), and it has also been reported to be present in heterochromatin (75).

We next performed *in vivo* experiments to validate the interaction of Crol with Cg and other PcG proteins. For this purpose, we first prepared polyclonal antibodies recognizing the N- or C-terminal parts of Crol, respectively (see Materials and Methods), both antibodies were confirmed to be IP grade in IP/Western-blot assay (Figure 1B; Supplementary File 2). To determine the interaction between Cg and Crol, we performed co-IP/Western blot assay for these two proteins. In support of our Y2H assay, Crol co-purified two major Cg bands (72) but not the IgG of non-immunized rabbit (Figure 1B). We further performed western-blot assays with Crol co-IPs against the PRC1 subunit Ph, PRC2 subunit E(z), and PRE-binding protein Pho, part of the PhoRC complex. In case of Ph, antibodies recognized both paralogs, Ph-p and Ph-d (72). Our data showed that Ph isoforms as well as Pho protein, but not E(z) were immunoprecipitated by Crol antibodies (Figure 1B). Crol's interactions with Cg and subunits of PRC1 and PhoRC complexes led us to hypothesize that Crol may play a role in PcG recruitment.

### Crol binds to PRE-elements *in vivo* and colocalizes with PcG proteins genome-wide

**Figure 2.**
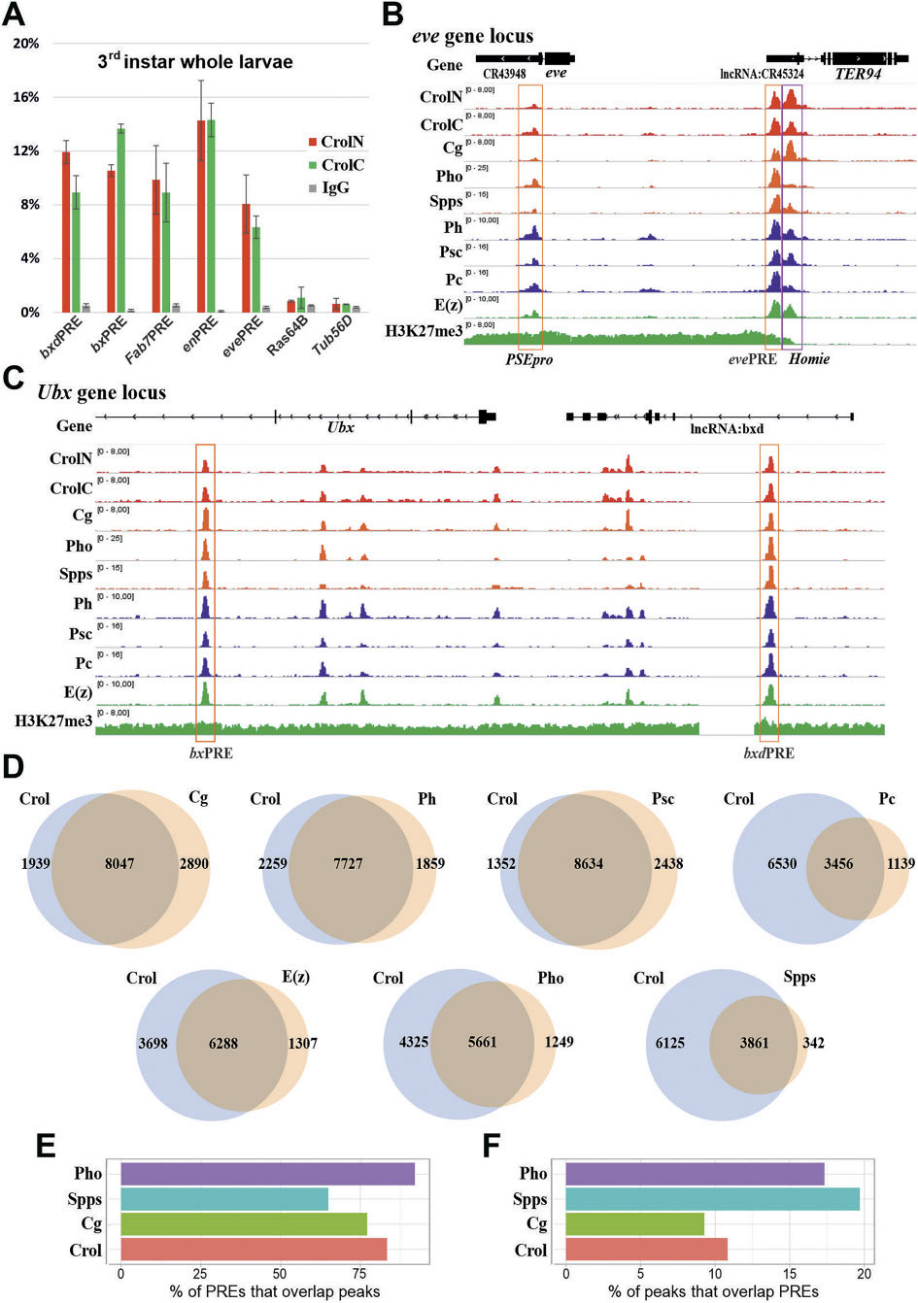
Crol colocalizes with PcG proteins and PREs genome-wide. (**A**) Crol enrichment at well-characterized PREs. X-ChIP with CrolN or CrolC antibody, or with IgG from a non-immunized rabbit as a negative control. The X-ChIP experiments were performed with chromatin isolated from the wild-type Oregon 3^rd^ instar whole larvae. The ordinate shows the percentage of target sequences in the immunoprecipitated material relative to the input DNA. X-ChIP was analyzed by real-time qPCR with primers specific to the PREs: *bxd*PRE, *bx*PRE, *Fab7*PRE, *en*PRE, *eve*PRE, or to negative genome controls: the coding part of Ras64B and Tubulin56D genes (indicated on the abscissa). All ChIP experiments were made in biological triplicate, vertical lines indicate SDs. (**B**) ChIP-seq profiles visualized as IGV tracks for Crol (CrolN and CrolC antibodies), Cg, Pho, Spps, Ph, Psc, Pc, E(z) and H3K27me3 histone modification. Chromatin was isolated from 3^rd^ instar wild-type larval brains and imaginal discs. The *even-skipped (eve)* gene regulatory domain is shown. The PSEpro proximal PRE and distant *eve*PRE located near the *TER94* gene are highlighted by orange boxes. The Homie-element that is also bound by PcG proteins is highlighted by a purple box. (**C**) The *Ubx* gene regulatory domain. The *bx*PRE and *bxd*PRE are highlighted by orange boxes. Other designations as in (B). (**D**) Venn diagrams showing the overlap between Crol and Cg, Ph, Psc, Pc, E(z), Pho and Spps. (**E**) Percent of PREs with Crol, Cg, Spps or Pho peaks. PREs were defined as regions with simultaneous binding of E(z), Ph, Pc and the presence of H3K27me3 histone modification. (**F**) Percent of Crol, Cg, Spps or Pho peaks within the PREs.

To test whether Crol binds to PREs, we first performed ChIP-qPCR at third instar whole larvae (Figure 2A) and embryo (Supplementary File 6, Figure S3). Crol showed enriched binding to several characterized PREs, including *bxd*PRE, *bx*PRE, *Fab7*PRE, *en*PRE2 and *eve*PRE, but not the coding sequences of the Ras64B and Tub56D genes. Therefore, Crol is a *bona fide* PRE-binding protein.

We applied ChIP-seq to determine the genome-wide occupancy of Crol in brains and imaginal discs of the third instar larvae. Since the two antibodies produced consistent results (Supplementary File 6, Figure S4), only data generated with the CrolN antibody were used for further analysis. We also performed ChIP-seq for E(z), Ph and H3K27me3, and collected public data for Pc, Psc, Pho, Spps and Cg (29,39). We first inspected several well-characterized PREs. Within the *even-skipped* (*eve*) gene domain, Crol co-binds with PcG proteins at the distant *eve*PRE adjacent to the TER94 promoter and the proximal PRE (i.e. PSEpro) adjacent to *eve*PRE promoter (76) (Figure 2B). The co-binding of Crol with PcG proteins is also evident at the *bx*PRE and *bxd*PRE of the *Ubx* locus (77) (Figure 2C), and all characterized PREs of the *Abd-B* and *invected-engrailed* loci (Supplementary File 6, Figure S5). Overall, Crol showed the highest degree of co-localization with Cg, Psc and Ph (Figure 2D). For example, the majority (81%) of Crol peaks are bound by Cg, and *vice versa*. Although lower percentages of Crol peaks overlap other PcG proteins, they still constitute > 75% of the peaks for Pc, E(z), Pho and Spps. Together, these data confirmed the binding of Crol to canonical PREs, and uncovered the global co-localization between Crol and PcG proteins.

We next determined the genome-wide binding of Crol to PREs, which were defined as H3K27me3(+) regions co-bound by E(z), Ph and Pc (Figure 2E). Crol is present at 80% of PREs in brains and imaginal discs of the wild-type 3^rd^ instar larvae. Remarkably, this is comparable to that estimated for Cg, Spps and Pho (Figure 2E). According to previous studies (29,31,78,79), core PRC1/2 subunits and PcG recruiters (i.e. Pho, Spps and Cg) frequently bind outside of H3K27me3 domains. Consistently, we found that 84% of Crol peaks are outside of H3K27me3 domains (Supplementary File 6, Figure S6). PREs constitute about 10% of Crol peaks, which is comparable to other PcG recruiters including Pho, Spps and Cg (Figure 2F).

Given that larvae brains and discs are made of mixed cell populations, their data are insufficient to validate the simultaneous binding of Crol and other PcG proteins at the same targets in the same cells. To overcome this limitation, we examined the overlap of Crol with PcG proteins and H3K27me3 modification using the S2 cell line as a model, which represents a more homogeneous system. A similar degree of overlap between Crol and PcG proteins was observed in S2 cells (Supplementary File 6, Figure S7): 66%, 42% and 66% of Crol peaks overlapped with Cg, Pc, and E(z) and they constituted 95%, 75% and 74% of Cg, Pc and E(z) peaks, respectively. Crol and Cg were present at 78% and 58% of PREs, respectively. At the same time these corresponded to only about of 3% of each of the Crol and Cg proteins peaks. The lower percentage of Crol and Cg peaks corresponding to PREs is due to lower number of H3K27me3(+) loci in S2 cells, indicating that a lower percentage of active PREs exist in S2 cells in comparison to third instar larvae brains and imaginal discs.

### Crol is a C2H2-type zinc finger protein that binds poly(G)-rich sequences

**Figure 3.**
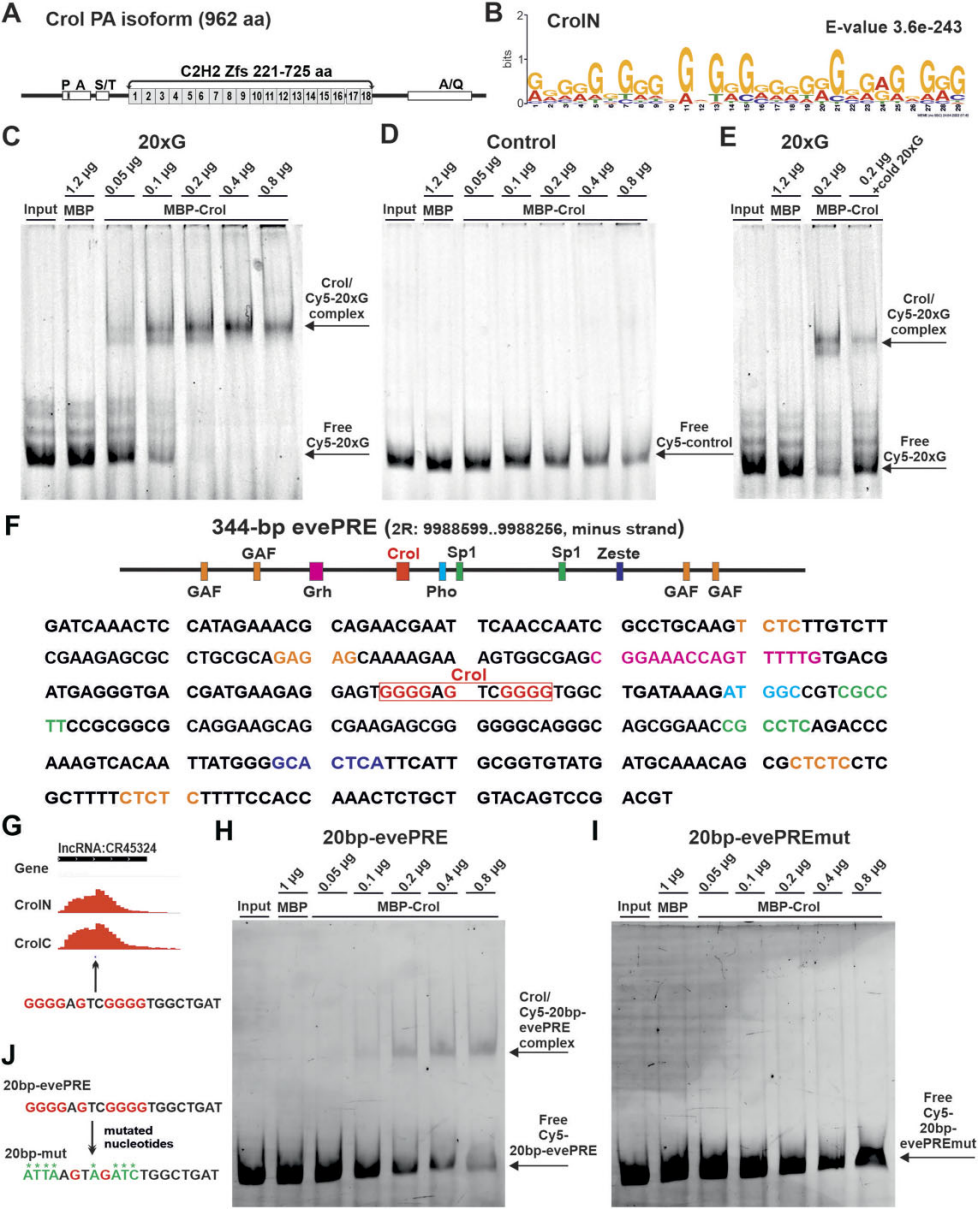
Crol is a C2H2-ZFP that binds poly(G)-rich sequences. (**A**) Structure of the Crol 962 aa PA isoform. Grey boxes—C2H2-type Zinc Finger motifs. (**B**) Motif identified at Crol sites using ChIP-seq. The ChIP-seq data obtained on third instar larval brains and imaginal discs with CrolN antibodies was used. (**C**) EMSA was performed with 20xG containing fragment and different amounts of Crol protein (see Materials and Methods). Probe order: 1 - Input – labeled DNA fragment without protein; 2 - labeled DNA fragment + MBP protein (1.2 μg); 3–7 - labeled DNA fragment + MBP-Crol fusion protein in the following amount (0.05, 0.1, 0.2, 0.4, 0.8 μg). The Crol/Cy5-20xG complex - complex between Crol protein and labeled 20xG containing fragment. (**D**) Negative control—the same DNA fragment as in (C) without 20xG was used. Order of probes the same as in (C). (**E**) Binding of Crol to 20xG containing fragment in the presence cold (unlabeled) competition (5 × molar excess). (**F**) The diagram of the DNA-binding sites and sequence of 344 bp *eve*PRE. The binding sites for the known PRE-binding proteins are shown according to (76): GAF (orange), Zeste (blue), Sp1 (green), Grh (purple), Pho (light blue), and predicted Crol-binding site (red). (**G**) Predicted Crol-site is centered on the CrolN and CrolC binding peak in *eve*PRE. (H, I) EMSA was performed with DNA fragment containing 20 bp-*eve*PRE (**H**) or with its mutated variant 20 bp-evePREmut (**I**). The same conditions as in (C) were used. (**J**) The nucleotides mutated in 20 bp-evePREmut DNA containing fragment are green colored.

The central part of the Crol protein is composed of 18 tandem C2H2-type zinc fingers (Figure 3A), which were reported to bind poly(G) sequences *in vitro* in a bacterial one-hybrid system (https://mccb.umassmed.edu/ffs/TFdetails.php?FlybaseID=FBgn0020309) (80). More specifically, bioinformatic prediction (63) and detailed alignment suggest that its poly(G)-binding specificity could be conferred by zinc fingers 4–15 (Supplementary File 6, Figure S8).

To assess the binding preference exhibited by Crol *in vivo*, we performed *de novo* motif discovery using the Crol ChIP-seq data for third instar wild-type larval brains and discs (Figure 3B, Supplementary File 6, Figure S8). As expected, a prominent yet unstructured G-rich motif is significantly enriched, which occurs in the majority of top-scoring Crol peaks (Figure 3B, Supplementary File 6, Figure S8). A similar motif was identified using ChIP-seq data on S2 cells (Supplementary File 6, Figure S8). These data suggest that *in vivo* Crol might recognize more variable poly(G)-rich motifs than suggested by the *in vitro* studies.

Our EMSA assay further confirmed that Crol binds to a synthesized DNA fragment containing 20xG motif instead of the non-specific control (Figure 3C, D). Moreover, cold 20xG competes with Cy5-20xG for Crol binding (Figure 3E). As an example of a natural Crol-binding site, we selected the poly(G)-rich motif present in the well-characterized 344-bp *eve*PRE which is adjacent to the TER94 gene promoter (76) and is bound by Crol *in vivo* (Figure 2B). This poly(G)-rich motif in *eve*PRE (Figure 3F) is centered on the Crol peak (Figure 3G). For the EMSA assay, we used DNA fragments containing 20 bp wild-type Crol-site (20bp-evePRE, Figure 3H) or its mutated variant (20 bp-evePREmut, Figure 3I, J). As expected, the binding of Crol was detected to the DNA fragment containing 20bp-evePRE but not to the 20 bp-evePREmut (Figure 3H, I).

### Crol is required for *eve*PRE dependent silencing in transgenes

**Figure 4.**
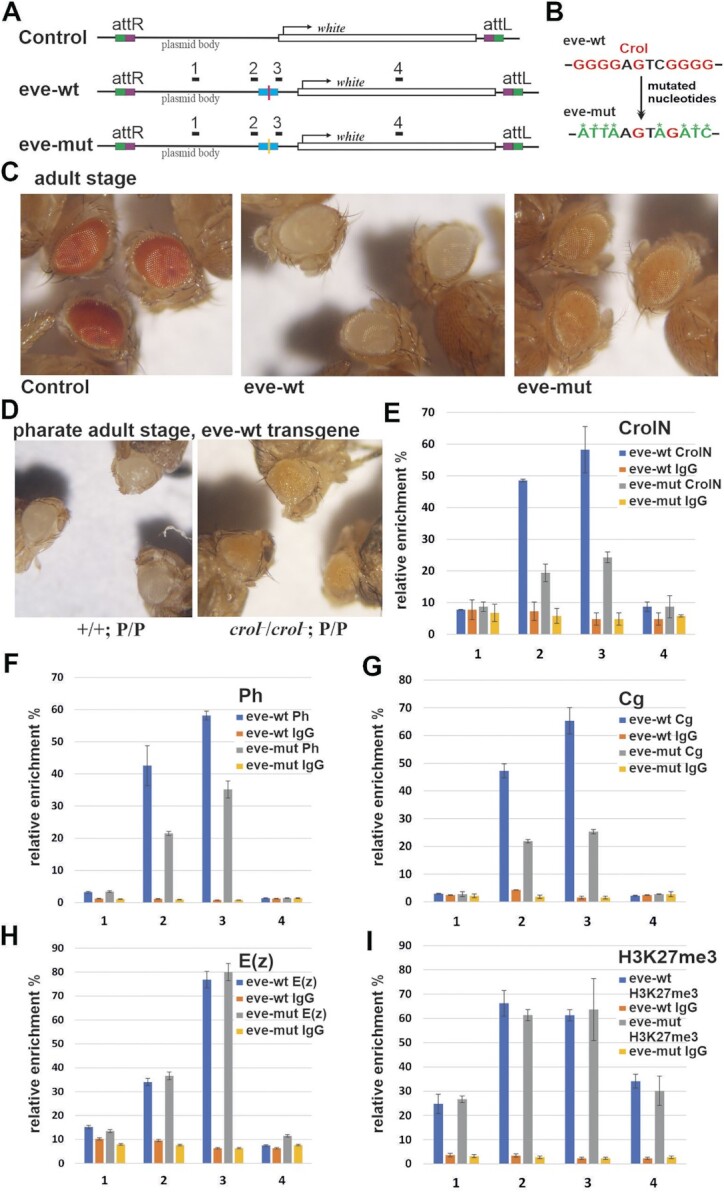
Crol is required for *eve*PRE dependent silencing in transgenes. (**A**) Diagram of transgene constructs after integration into the genome. Control construct has *white* reporter gene, attR and attL. attR and attL are result of recombination between attB and attP at insertion site. eve-wt in addition has wild-type 344 bp *eve*PRE, eve-mut—344 bp *eve*PRE with mutated Crol-binding sites. Numbers on top of the construct schemes 1, 2, 3 and 4 indicate regions amplified by qPCR in X-ChIP experiments. The transgenes were integrated into the attP2 integration site. (**B**) The nucleotides mutated in 344 bp eve-mut were the same as in 20 bp-mut tested in EMSA in Figure 3J. (**C**) The eye phenotypes of homozygous adult stage flies that have the control, eve-wt or eve-mut transgenes are shown. (**D**) The eye phenotypes of homozygous pharate adult stage eve-wt control flies (+/+; P/P, left) or eve-wt flies with *crol*-KO background (*crol*^−^/*crol*^−^; P/P, right) are shown. (E–I) The ChIP experiments were performed with chromatin isolated from 3^rd^ instar wild-type larval homozygous for the eve-wt or eve-mut transgenes. The X-ChIPs were performed with specific antibodies or with IgG. The specific antibodies: CrolN (**E**), Ph (**F**), Cg (**G**), E(z) (**H**) and H3K27me3 (**I**). The ordinate shows the percentage of target sequences in the immunoprecipitated material relative to the input DNA and normalized to the positive control—a sequence adjacent to the endogenous *bxd*PRE in BX-C (*bxd*PRE-Genome). The transgene specific regions 1, 2, 3 and 4 are indicated on the abscissa. All ChIP experiments were done in biological triplicate, vertical lines indicate SDs.

We next tested the role of the Crol motif in *eve*PRE activity in transgenes. The following transgene constructs were made (Figure 4A): (i) the Control construct, carried the *white* reporter gene responsible for red-colored eye pigmentation and the attB site required for site-specific integration using the PhiC31 system (71); (ii) the eve-wt construct, had an insertion of the wild-type 344-bp *eve*PRE; (iii) the eve-mut construct, carried the 344-bp *eve*PRE with the same mutation of Crol site as was tested in EMSA (Figure 4B). The transgene constructs were inserted in the attP2 site in flies lacking a functional *white* gene (71). The silencing activity of the *eve*PRE was assessed by the reduction in eye pigmentation, which is known to be directly correlated with the level of *white* gene transcription (81,82). Figure 4C shows the eyes of homozygous flies for each construct. The flies carrying the Control construct had red-colored eyes indicating active *white* gene transcription. In contrast, flies with the wild-type *eve*PRE have white-colored eyes indicating complete repression of the *white* gene. Importantly, flies containing the *eve*PRE with mutation in poly(G)-rich motif are more pigmented, indicating partial release of silencing (Figure 4C).

To further address the function of Crol at PREs, we created *crol* knockout (*crol*-KO) flies by CRISPR/Cas9 technology (Supplementary File 6, Figures S1, S2). The homozygotes of the *crol-*KO flies died mostly at the pharate adult stage with a ‘crooked leg’ phenotype (not shown) in accordance with previous data (83). We examined the effect of *crol-*KO mutants on transgenic flies carrying eve-wt construct. Figure 4D shows eyes of pharate adults homozygous for the **eve-wt** construct with (*+/+;* P/P) and without *crol* (*crol*^−^/*crol*^−^; P/P). Consistent with a role of Crol in PRE-mediated repression of transcription, in the absence of *crol*, the silencing of the *white* reporter was suppressed. Similar results were obtained with another transgene carrying a 656-bp *bxd*PRE. Upon *crol-*KO repression of the *white* gene by *bxd*PRE was reduced (Supplementary File 6, Figure S9). Thus, Crol is required for PRE-mediated repression in transgenes.

We next performed the ChIP-qPCR on the third instar whole larvae bearing eve-wt or eve-mut transgenes and examined the impact of Crol site mutation on Crol and PcG binding. ChIP was performed for Crol, Ph, Cg, E(z) and H3K27me3. As a negative control, the ChIP was performed against PRE-binding protein GAF which failed to directly interact with Crol (Supplementary File 1), and we expected that its binding to be independent of Crol. The results demonstrate that mutation of the Crol site in the *eve*PRE affected the binding of Crol (Figure 4E), Ph (Figure 4F) and Cg (Figure 4G), but had no effect on the enrichment of E(z) (Figure 4H), GAF (Supplementary File 6, Figure S10), or H3K27me3 (Figure 4I). Thus, Crol is involved in the recruitment of Ph and Cg to the transgenic *eve*PRE.

### *Knockout of crol* leads to decreased occupancy of Ph and Cg genome-wide

We next determined the genome-wide dependency of PcG binding on Crol by performing paralleled ChIP-seq in wild-type (WT) and *crol*-KO third instar larval brains and discs. Despite the apparent decrease of Crol protein upon *crol*-KO by Western-blot assay (Supplementary File 2), we only identified 1170 genomic loci with significantly decreased Crol binding (Supplementary File 6, Figure S11). This suggests that in *crol*-KO flies, part of maternally provided Crol protein remains stably associated with its targets throughout development. Accordingly, only these 1170 Crol-decreased loci were examined for differential binding analysis of PcG proteins. Upon *crol-*KO, the binding of Ph and Cg was decreased at 346 and 72 of peaks respectively, but no significant changes were seen for E(z) or H3K27me3 (Supplementary File 6, Figure S11). These data are consistent with the results obtained by mutating Crol binding sites in the *eve*PRE-transgene (above). The coordinates of differential binding peaks for Crol, Ph and Cg are given in Supplementary File 7.

**Figure 5.**
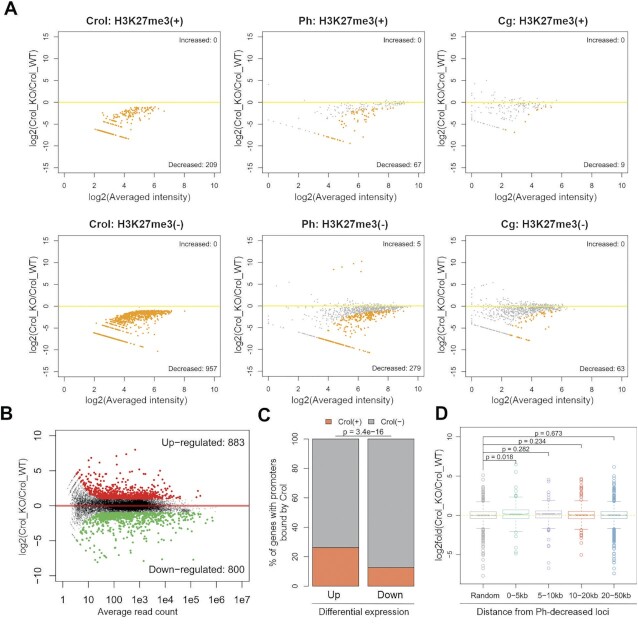
Knockout of *crol* affects the Ph and Cg binding genome-wide and dysregulates genes transcription. (**A**) The MA-plots show the changes in the binding of the Crol, Ph, and Cg in *crol*-KO vs wild-type third instar larval brains and imaginal discs at the sites at which Crol binding is decreased upon *crol* knockout. The H3K27me3+ (upper) and H3K27me3- (lower) genomic regions were analyzed separately. Peaks showing significant differential binding (FDR < 0.05) as identified by DiffBind are marked in orange**. (B**) The MA-plot shows the change in RNA-seq profile upon *crol*-KO vs wild-type third instar larvae. Significantly up- and down-regulated genes are highlighted in red and green, respectively. (**C**) Barplot shows the percentages of genes with Crol binding within ±1 kb of their TSSs. The two groups of genes with up- or down-regulated expression after *crol*-KO) were analyzed separately. *P*-values calculated by Fisher's exact test is indicated. (**D**) Comparison of the altered expression of different groups of genes stratified by their distance to loci with decreased Ph-binding. Four groups of genes were defined, with their expression changes compared against 1000 randomly selected genes. The *P*-values calculated by using Two-sided Student's t-test were indicated. Only genes near Ph-decreased loci showed significantly different changes in gene expression.

Given that Crol binds to not only PREs but also numerous non-repressed genome loci, we separately examined H3K27me(+) and H3K27me(−) regions, which contain 209 and 957 Crol peaks decreased upon *crol-*KO, respectively (Figure 5A). Both H3K27me(+) and H3K27me(−) domains contained peaks that showed significant decrease of Ph (32% and 29.1% respectively) and Cg (4.3% and 6.6% respectively) binding upon *crol-*KO (Figure 5A).

To ask whether *crol-*KO affects transcription of *ph* or *cg*, we performed RNA-seq using *crol-*KO and wild-type third instar larvae. The results showed that *crol-*KO does not reduce the transcription of *ph (ph-d and ph-p)* or *cg*. Instead, we observed slightly increased transcription of both genes (Supplementary File 6, Figure S11), probably because they are under PcG-mediated repression. In agreement, this was previously demonstrated for *ph* (84).

Thus, Crol is involved in the genome-wide recruitment of Ph and Cg in both H3K27me3(+) and H3K27me3(−) domains.

### Crol regulates transcription of the target genes

We next analyzed the impact of *crol*-KO on gene transcription by performing RNA-seq analysis. While a significant amount of Crol protein is still present in larvae, we identified 1683 genes with altered expression upon *crol-*KO, including 883 up-regulated and 880 down-regulated (Figure 5B, Supplementary File 8). GO analysis demonstrated that up-regulated genes are associated with neuron-related functions, while down-regulated genes are associated with mannose metabolic process and infection response (Supplementary File 6, Figure S12). Importantly, previous studies have showed that up-regulated genes in *Spps* and *pho* mutants are both enriched for neurogenesis, while down-regulated genes include those that are associated with infection response (29). Thus, the effect of *crol-*KO on the transcriptome is very similar to the effects observed upon mutation of *pho* and *Spps*.

To uncover the potential link between Crol and transcriptional repression, we further examined the binding of Crol on each group of the differentially expressed genes. Interestingly, 26.2% of up-regulated genes have Crol binding within +/- 1kb of their TSSs, which is significantly higher than down-regulated genes of which only 12.6% were bound by Crol (Figure 5C). We further compared the altered expression of different gene groups stratified by their distance to Ph-decreased loci after *Crol*-KO, and found that genes with decreased Ph-binding within 5 kb to their TSSs tend to have increased expression after *crol*-KO (Figure 5D). These results indicate that Crol has repressive effect over their target genes, likely via mediating the recruitment of Ph and probably some other PcG proteins.

**Figure 6.**
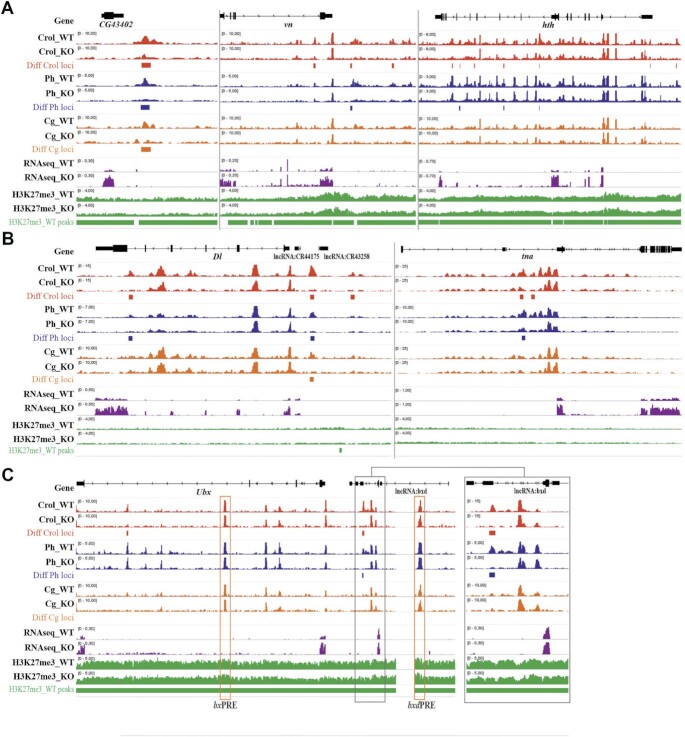
Effects of *crol*-KO at representative gene regions. The IGV tracks demonstrate the differential binding of Crol, Ph and Cg, and upregulation of target genes (RNAseq data) at the representative gene regions in wild-type (WT) and *crol*-KO (KO) 3^rd^ instar larval brains and imaginal discs. The H3K27me3 tracks and peaks are shown to designate H3K27me3(+) gene domains. (**A**) *CG43402, vn*, and *hth* genes are in H3K27me3(+) domains. (**B**) *Dl* and *tna* genes are in H3K27me3(−) domains. (**C**) *Ubx* gene is in H3K27me3(+) domain. Known PREs are highlighted by orange boxes. The region where binding of Crol and Ph is affected by the *crol-*KO is in the black box, enlarged in the right side of the diagram.

Closer inspection identified loci at which *crol*-KO leads to decreased Crol/Ph binding and coincidently increased transcription. For example, the *CG43402*, *vn*, and *hth* genes are within H3K27me3(+) domain (Figure 6A), while *Dl* and *tna* are not (Figure 6B). Of note, the *CG43402* and *Dl* loci also demonstrate remarkably decreased Cg binding. In most cases, the significantly decreased Crol/Ph/Cg peaks are regulatory regions of upregulated genes. We propose that the disbalance of Crol binding to the associated regulatory regions is sufficient for the derepression of the target genes. Of well-characterized PREs from *eve, invected-engrailed* and *Abd-B*, we detected significantly decreased Crol binding only on *Fab6*PRE, yet without significantly altered Ph/Cg binding or *Abd-B* transcription (not shown). Meanwhile, while the Crol/Ph binding wasn’t affected at the *bx-* and *bxd*PREs, we detected significantly increased transcription of *Ubx* gene upon *crol-*KO (Figure 6C). This correlated with significantly decreased binding of Crol and Ph upstream of the *Ubx* promoter (Figure 6C). We suggest that this region might be an uncharacterized regulatory element responsible for observed *Ubx* gene activation upon *crol*-KO.

Thus, Crol controls the transcription of many genes and can repress gene expression both inside and outside of H3K27me3 domains.

## DISCUSSION

Here, we have identified Crol as a new PcG recruiter. We show that Crol is a direct Cg protein partner and using co-immunoprecipitation experiments, we demonstrate that in addition to Cg, Crol interacts with Ph of the PRC1 complex and with the PRE-binding protein Pho. Using ChIP-seq analysis, we demonstrate that Crol colocalizes with PcG proteins genome-wide, including the classical PREs of the *eve*, *en*, *Ubx* and *Abd-B* gene domains.

Crol is a C2H2-ZFP that binds to poly(G)-rich sequences both *in vitro* and *in vivo* ((80) and this paper). In a transgene assay, we demonstrate that Crol-bound poly(G)-rich motif is important for proper *eve*PRE-mediated silencing and for recruitment of Ph and Cg proteins to *eve*PRE. Moreover, *crol*-KO reduces the ability of the *eve*PRE and *bxd*PRE to silence the *mini-white* gene in the transgenes.

Due to maternally deposition of Crol protein and its stable association with many chromatin sites throughout development, we were able to obtain only reduced levels of Crol upon it's KO. Decreased levels of Crol were sufficient to affect binding of Crol/Ph/Cg to transgenic *eve*PRE, but not to the endogenous *eve*PRE. These can reflect the cooperative nature of PREs that support each other in the endogenous context and promotes the binding of Crol to PREs in its natural genome environment.

In total, Crol binding in Crol KO flies was decreased at 1170 of genome sites and of them Ph and Cg were decreased at 346 and 72 of sites, respectively. Crol function extends beyond genes within H3K27me3 domains and Ph/Cg are affected at proportionally equal Crol decreased peaks in H3K27me3+ and H3K27me3- domains. The similar importance for Ph recruitment outside of H3K27me3 domains was previously reported for the Cg protein (39).

The lower sensitivity of Cg than Ph to decrease of Crol level suggests that Crol is not a bona fide Cg recruiter, and its role in Cg recruitment is highly dependent upon local content. This may reflect the combinatorial nature of Ph-associated regulatory elements with different degrees of participation of a particular DNA-binding factor in Ph recruitment to distinct targets.

Importantly, *crol*-KO leads to inappropriate expression of 1683 genes genome-wide, of which 883 genes are upregulated, indicating that partial loss of Crol protein is sufficient to disbalance gene transcription. The dysregulation of gene transcription correlates with loss of Crol/Ph peaks at corresponding loci but doesn’t require dissociation of Crol from all peaks in the loci.

For example, in the case of the *Ubx* gene, *crol*-KO doesn’t affect the level of Crol/Ph protein at the well-defined *bx-* and *bxd*PREs but leads to a decreased Crol/Ph binding at an upstream gene region and correlates with an increase in *Ubx* gene transcription. Similarly, in the case of the *vn* and *Dl* genes, Crol/Ph are still bound to promoters of genes but are lost in regulatory regions of the corresponding genes.

Importantly, GO analysis indicates that *crol-*KO affects the transcription of same set of genes as does the mutation of two other PRE-binding proteins, Pho and Spps. In *crol*, *Spps* and *pho* mutants, neuron-related functions genes are up-regulated, and genes implicated in infection response are downregulated. This suggests that Crol, Pho, and Spps may co-regulate these genes.

The number of proteins implicated in PcG repressor recruitment is continually growing (22). Our results suggest that PcG factors can interact with a diverse array of DNA-binding proteins and that interactions create a combinatorial platform for the recruitment of PcG complexes at target sites in *Drosophila* genome.

## ETHICS APPROVAL

Animal handling for the antibody production was carried out strictly according to the procedures outlined in the NIH (USA) Guide for the Care and Use of Laboratory Animals. The protocols used were approved by the Committee on Bioethics of the Institute of Gene Biology, Russian Academy of Sciences. All procedures were performed under the supervision of a licensed veterinarian, under conditions that minimize pain and distress.

Rabbits were purchased from a licensed specialized nursery, Manihino. Soviet chinchilla rabbits used in the study are not endangered or protected. Only healthy rabbits, certified by a licensed veterinarian were used. The rabbits were individually housed in standard size, stainless steel rabbit cages and provided an *ad libitum* access to alfalfa hay, commercial rabbit food pellets, and water. The appetite and behavior of each rabbit was monitored daily by a licensed veterinarian. Body weight and temperature of each rabbit were evaluated prior to and daily following the immunization. No animals became ill or died at any time prior to the experimental endpoint. At the end of the study period all rabbits were euthanized by intravenous injection of barbiturate anesthetics.

## DATA AVAILABILITY

All relevant data are within the paper and its Supporting Information. The ChIP-seq and RNA-seq data generated in this study have been deposited in NCBI GEO database with accession GSE202872. The following publicly available ChIP-seq was used for Psc, Pho and Spps (GSE102339), Pc (GSE102339), Cg (GSE77582) (third instar larval brains and imaginal discs); Pc (GSE24521), H3K27me3 (GSE41440) and E(z) (GSE101554) (S2 cells).

## Supplementary Material

gkad336_Supplemental_Files

## Notes

Present address: Lika V Fab, Laboratory of Neurogenetics and Genetics Development, Institute of Higher Nervous Activity and Neurophysiology of Russian Academy of Sciences, Moscow, Russia.
