# Supplementary material for: Crol contributes to PRE-mediated repression and Polycomb group proteins recruitment in Drosophila
Source: Nucleic Acids Res. 2023 May 4;51(12):6087–100. doi: 10.1093/nar/gkad336 (PMC10325914; doi:10.1093/nar/gkad336)
Supplement: gkad336_Supplemental_Files [file gkad336_supplemental_files.zip › Erokhin et al - Supplementary File 2. Western-blot analysis.pdf]

Supplementary file 2

Erokhin et al.

Crol contributes to PRE-mediated repression and Polycomb group proteins recruitment in *Drosophila*

This file includes Supplementary Figures S2-1- S2-7.

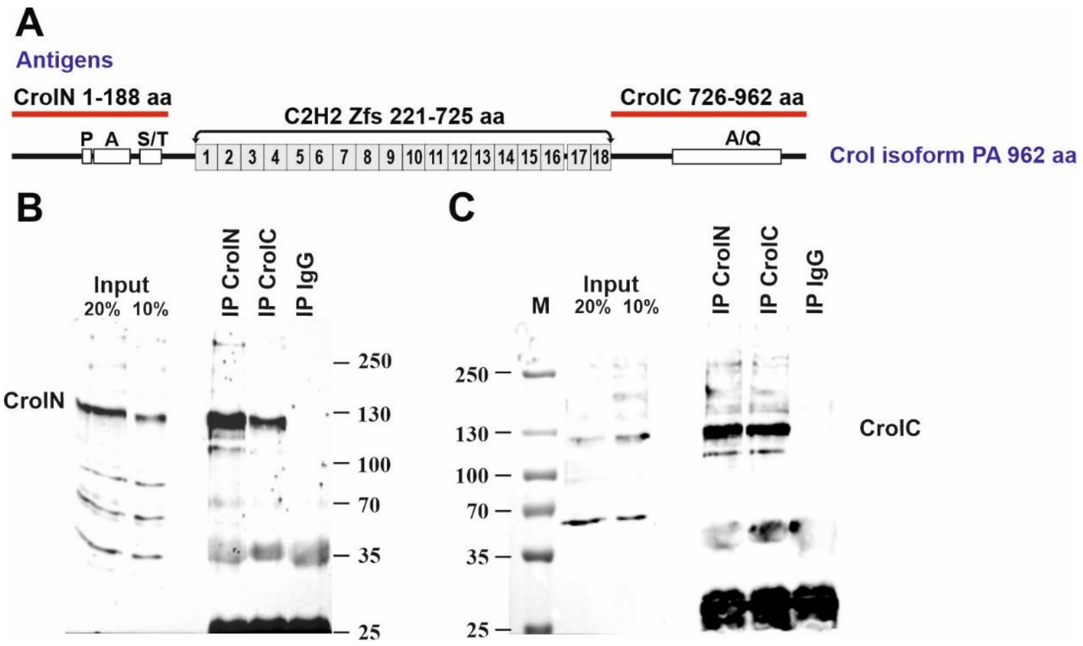

Supplementary Fig. S2-1. Crol antibodies specificity test by IP/Western-blot. **A**) Structure of Crol protein. Antigens for CrolN and CrolC antibodies are indicated. **B, C**) S2 *Drosophila* cell nuclear extracts were incubated with α-CrolN (CrolN), α-CrolC (CrolC) rabbit antibodies, or IgG of non-immunized rabbit (IgG). Lysates (Input), precipitated fractions (IP) with Crol specific antibodies or with IgG were Western blotted with antibodies against CrolN (**B**) or CrolC (**C**).

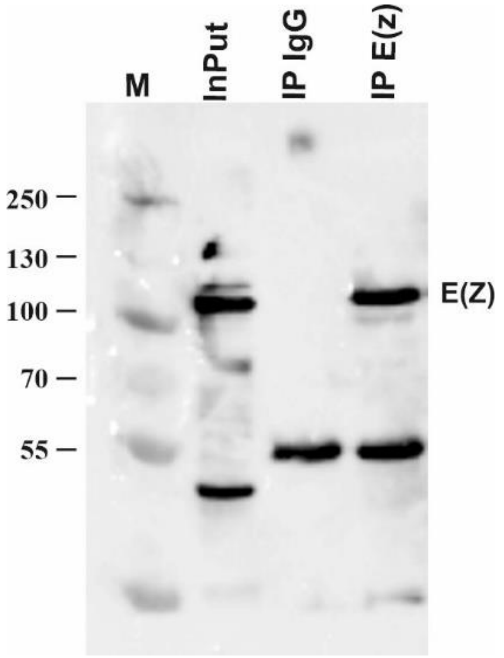

Figure S2-2. E(z) antibodies specificity test by IP/Western-blot. S2 *Drosophila* cell nuclear extracts were incubated with α-E(z) rabbit antibodies, or IgG of non-immunized rabbit (IgG). Lysates (Input), precipitated fractions (IP) with E(z) specific antibodies or with IgG were Western blotted with antibodies against E(z)**.**

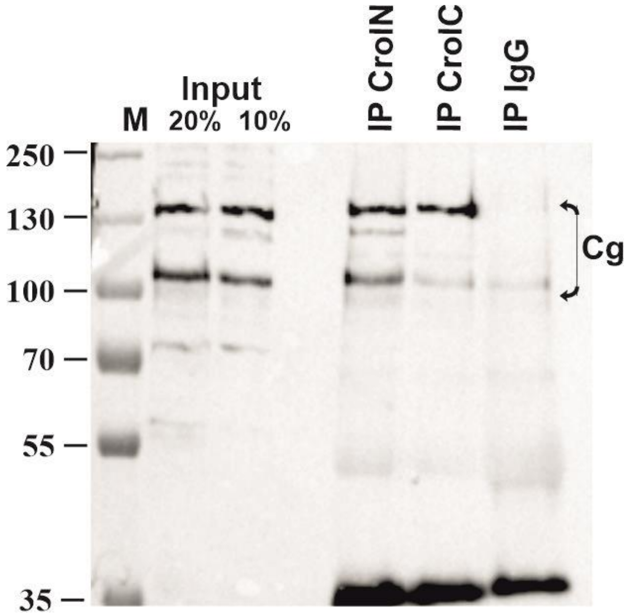

Figure S2-3. Western-blot analysis of Crol co-IP with Combgap. S2 *Drosophila* cell nuclear extracts were incubated with α-CrolN (CrolN), α-CrolC (CrolC) rabbit antibodies, or IgG of non-immunized rabbit (IgG). Lysates (Input), precipitated fractions (IP) with Crol specific antibodies or with IgG were Western blotted with antibodies against Combgap**.**

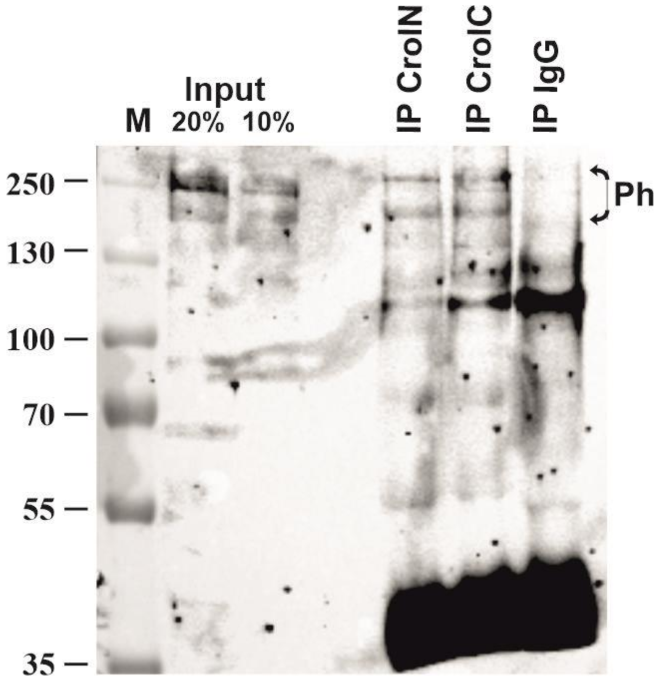

Figure S2-4. Western-blot analysis of Crol co-IP with Ph. S2 *Drosophila* cell nuclear extracts were incubated with α-CrolN (CrolN), α-CrolC (CrolC) rabbit antibodies, or IgG of non-immunized rabbit (IgG). Lysates (Input), precipitated fractions (IP) with Crol specific antibodies or with IgG were Western blotted with antibodies against Ph.

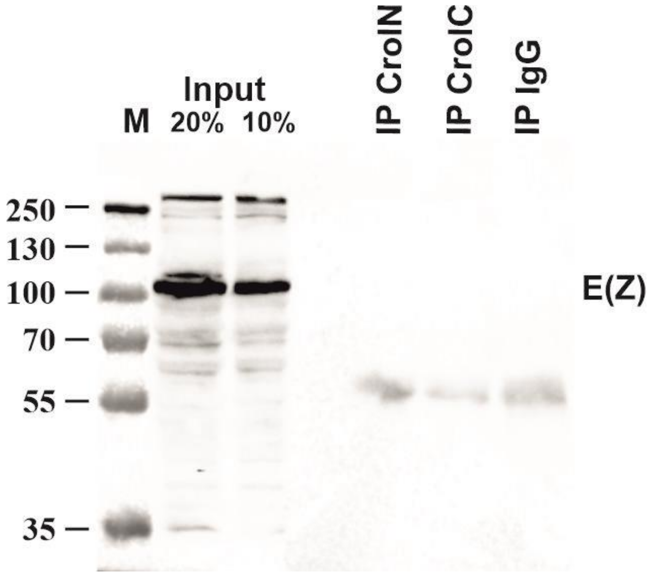

Figure S2-5. Western-blot analysis of Crol co-IP with E(z). S2 *Drosophila* cell nuclear extracts were incubated with α-CrolN (CrolN), α-CrolC (CrolC) rabbit antibodies, or IgG of non-immunized rabbit (IgG). Lysates (Input), precipitated fractions (IP) with Crol specific antibodies or with IgG were Western blotted with antibodies against E(z).

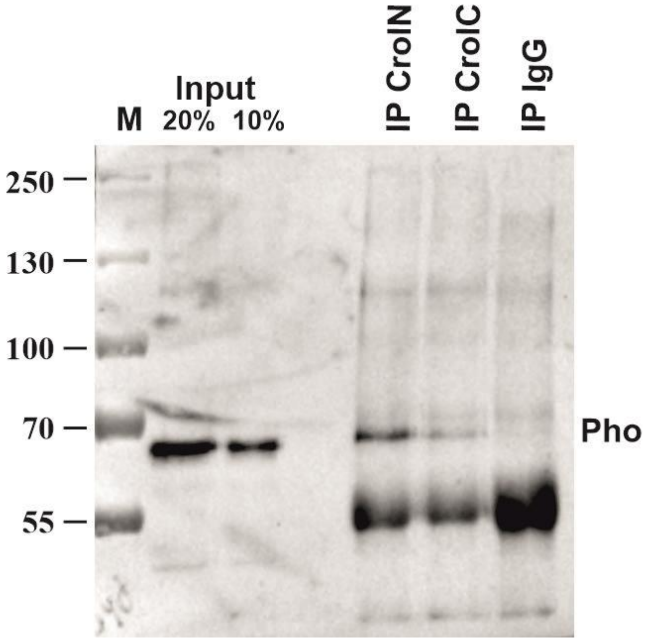

Figure S2-6. Western-blot analysis of Crol co-IP with Pho. S2 *Drosophila* cell nuclear extracts were incubated with α-CrolN (CrolN), α-CrolC (CrolC) rabbit antibodies, or IgG of non-immunized rabbit (IgG). Lysates (Input), precipitated fractions (IP) with Crol specific antibodies or with IgG were Western blotted with antibodies against Pho.

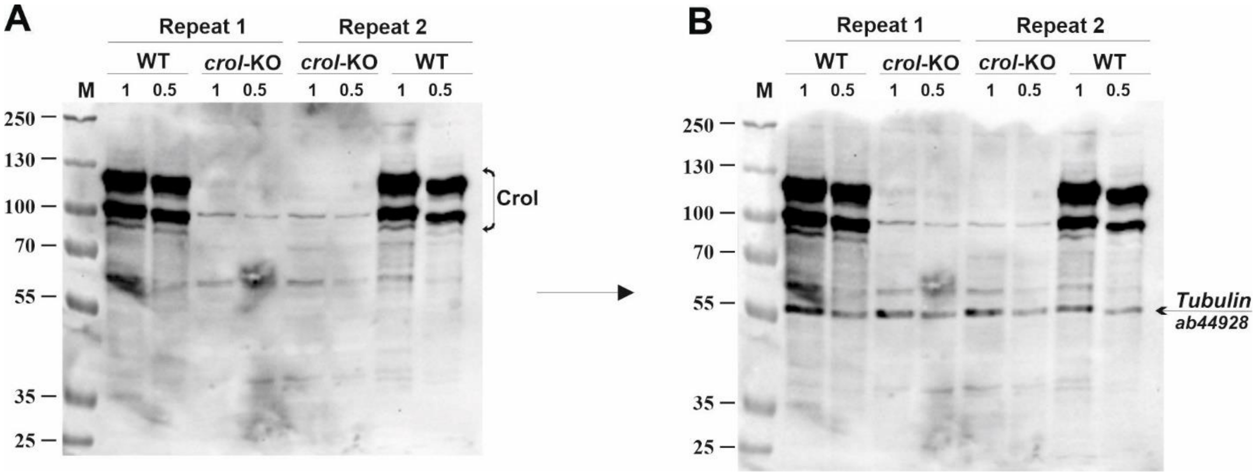

Figure S2-7. Examination of Crol levels in *crol*-KO mutants. **A**) Nuclear extracts from wild-type (WT) and *crol*-KO 3rd instar wild-type larval imaginal discs and brains probed with anti-CrolN antibody. Note that light bands of Crol are visible in the *crol*-KO lines. We suspect this bands represents maternally deposited Crol protein. **B**) Control-blot performed over the Crol-blot in (A) with anti-Tubulin antibody.
