# Supplementary material for: Crol contributes to PRE-mediated repression and Polycomb group proteins recruitment in Drosophila
Source: Nucleic Acids Res. 2023 May 4;51(12):6087–100. doi: 10.1093/nar/gkad336 (PMC10325914; doi:10.1093/nar/gkad336)

Supplementary file 4

Erokhin et al.

Crol contributes to PRE-mediated repression and Polycomb group proteins recruitment in *Drosophila*

Supplementary Material and Methods

This file describes the following methods:

- **Generation of Control, eve-wt and eve-mut constructs;**
- **Generation of constructs for *crol* knockout (KO) flies by CRISPR-Cas9–induced homologous recombination;**
- **ChIP (Chromatin immunoprecipitation)-qPCR;**
- **ChIP-seq;**
- **Sequences of DNA-fragments obtained by PCR for EMSA.**

I. Generation of Control, eve-wt and eve-mut constructs

The 344-bp *eve*PRE (PCR-amplified with primers 5′-acgtcggactgtacagcagag-3′ and 5′-gagcaaactccatagaaacgcag-3′) was cloned into pBluescript SK+ vector and sequenced to confirm its identity (*eve*PRE-pSK plasmid).

The 344-bp *eve*PREmut was created via PCR-mutagenesis (*eve*PREmut-pSK plasmid). The accuracy of the replacement was confirmed by sequencing.

The *Bam*HI-*Eco*RI fragment containing the white gene (*mini-white* version) without Wari insulator was clones from pCaSpeRΔ700 vector (Chetverina et al, NAR, 2008) into the pBluescript SK+ vector to obtain white-rev-pSK plasmid. The attB site was PCR-amplified with 5′-gtcgacgatgtaggtcacgg-3′ and 5′-gtcgacatgcccgccgtgac-3′ primers and cloned downstream of the *white* gene (**Control** construct, described as attBdir-white-rev-pSK plasmid previously) (Erokhin et al., BMC Biol, 2021).

To create **eve-wt** and **eve-mut** the *Xho*I-*Kpn*I fragment of the *eve*PRE or *eve*PREmut was cut out of *eve*PRE-pSK plasmid or *eve*PREmut-pSK plasmid, respectively, and inserted into the attBdir-

white-rev-pSK plasmid cleaved by *Pst*I and *Kpn*I. For the ligation, the corresponding samples after digestion *Xho*I or *Pst*I were treated by T4 DNA polymerase and only after digested by *Kpn*I.

References:

Chetverina D, Savitskaya E, Maksimenko O, Melnikova L, Zaytseva O, Parshikov A, Galkin AV, Georgiev P. Red flag on the white reporter: a versatile insulator abuts the white gene in Drosophila and is omnipresent in mini-white constructs. Nucleic Acids Res. 2008 Feb;36(3):929-37. doi: 10.1093/nar/gkm992. Epub 2007 Dec 17. PMID: 18086699; PMCID: PMC2241909.

Erokhin M, Gorbenko F, Lomaev D, Mazina MY, Mikhailova A, Garaev AK, Parshikov A, Vorobyeva NE, Georgiev P, Schedl P, Chetverina D. Boundaries potentiate polycomb response element-mediated silencing. BMC Biol. 2021 Jun 2;19(1):113. doi: 10.1186/s12915-021-01047-8. PMID: 34078365; PMCID: PMC8170967.

II. Generation of constructs for *crol* knockout (KO) flies by CRISPR-Cas9–induced homologous recombination

Generation of plasmid coding gRNA (CRISPRs) to *crol* gene

The flyCRISPR target finder online program (https://flycrispr.org/ , Gratz et al, Genetics, 2014) was used to select optimal guide RNA target sites (gRNA, CRISPRs) for the 5′ and 3′ end of the *crol* gene (**Supplementary File 6, Figure S1A, B**). The following *crol*-target CRISPRs were selected: 5′-crol CRISPR – 5′-aggtggtgtcacttagtctc-3′ and 3′-crol CRISPR – 5′-agcggatatcgtgatacgct-3′. The 5′- and 3′-crol CRISPRs were simultaneously cloned by a PCR-based method into the dual expression prAc_yiless_U6:1:3_attB vector. The 5′- and 3′-crol CRISPRs were cloned downstream U6:1 and U6:3 promoters, respectively. The prAc_yiless_U6:1:3_attB vector (Zolotarev et al, NAR, 2017) is based on pCFD4-U6:1_U6:3tandemgRNAs plasmid (Addgene # 49411, Port et, PNAS, 2014) with additional insertion of the *yellow*-intron-less gene under control of *Actin5C* promoter.

For simultaneous cloning 5′- and 3′-crol CRISPRs were incorporated into the forward and reverse primers, which also contain homology to prAc_yiless_U6:1:3_attB vector for PCR-amplification from this vector (**Supplementary File 6, Figure S1C**). In addition, primers contain BbsI sites and spacers for cloning.

The following primers were made:

1) *forward* with 5′-crol CRISPR – 5′-gagaagacctttcggAGGTGGTGTCACTTAGTCTCGTTTTAGAGCTAGAAATAGCAAG-3′;

2) *reverse* with 3′-crol CRISPR – 5′-gagaagacctaaacAGCGTATCACGATATCCGCTcGACGTTAAATTGAAAATAGGTC-3′.

The detailed structure of primers is shown on **Supplementary File 6, Figure S1C.**

The BbsI-digested PCR products were cloned into the prAc_yiless_U6:1:3_attB vector digested with BbsI. BbsI recognizes 5′-GAAGACN2↓-3′/3′-CTTCTGN6↑-5′ present in the 5′-parts of primer and in prAc_yiless_U6:1:3_attB vector. The scheme of result CRISPR-*crol*-target plasmid is shown of **Supplementary File 6, Figure S1D.** Correct ligation of the *crol* CRISPR target sequences was confirmed by sequencing.

Generation of HR-crol plasmid for homological recombination

The *crol*-homology arms surrounding the *crol*-CRISPRs target sites were PCR amplified from wild-type $y^{-}w^{1118}$ genomic DNA using the following primers:

1) 5′-AAGAATTCGTCATTCGTTTTGCTGCCTT-3′ and 5′-AACCGCGGAGGTATTTTTCGAGGTCTTT-3′ for the 5′-Crol homology arm (767-bp fragment),
2) 5′-TTAGATCTTCTAGGGTGTGTCGATTTT-3′ and 5′-AACTGCAGACTTTCAGGGCTCGATATA-3′ for the 3′-Crol homology arm (906-bp fragment) (**Supplementary File 6, Figure S1B).**

Next, the 5′- and 3′-*crol*-homology arms were cloned into the pDsRed-attP plasmid. This vector contains the dsRed gene under 3xPE promoter, flanked by lox sites and has attP site for φC31. pDsRed-attP was a gift from Melissa Harrison & Kate O'Connor-Giles & Jill Wildonger (Addgene plasmid # 51019; http://n2t.net/addgene:51019; RRID:Addgene 51019). The final plasmid contains genetic elements in the following order: [5′-Crol homology arm]-[attP]-[lox]-[3×P3-dsRed-SV40polyA]-[lox]-[3′-Crol homology arm] (**Supplementary File 6, Figure S1E**).

References:

Gratz SJ, Ukken FP, Rubinstein CD, Thiede G, Donohue LK, Cummings AM, O'Connor-Giles KM. Highly specific and efficient CRISPR/Cas9-catalyzed homology-directed repair in Drosophila. Genetics. 2014 Apr;196(4):961-71. doi: 10.1534/genetics.113.160713. Epub 2014 Jan 29. PMID: 24478335; PMCID: PMC3982687.

Zolotarev N, Maksimenko O, Kyrchanova O, Sokolinskaya E, Osadchiy I, Girardot C, Bonchuk A, Ciglar L, Furlong EEM, Georgiev P. Opbp is a new architectural/insulator protein required for ribosomal gene expression. Nucleic Acids Res. 2017 Dec 1;45(21):12285-12300. doi: 10.1093/nar/gkx840. PMID: 29036346; PMCID: PMC5716193.

Port F, Chen HM, Lee T, Bullock SL. Optimized CRISPR/Cas tools for efficient germline and somatic genome engineering in Drosophila. Proc Natl Acad Sci U S A. 2014 Jul 22;111(29):E2967-76. doi: 10.1073/pnas.1405500111. Epub 2014 Jul 7. PMID: 25002478; PMCID: PMC4115528.

III. ChIP (Chromatin immunoprecipitation)-qPCR

Chromatin immunoprecipitation (X-ChIP) was prepared as describe previously (Erokhin et al., BMC Biol, 2021). For each experiment, 150-200 mg of 3rd instar larvae or embryos were collected. The material was homogenized in 5 ml of buffer A1 (15 mM HEPES, pH 7.6; 60 mM KCl, 15 mM NaCl, 4 mM MgCl2, 0.5% Triton X-100, 0.5 mM DTT, Roche cOmplete protease inhibitor) supplemented with the EDTA-free protease inhibitor cocktail (Roche, Switzerland) and formaldehyde as a crosslinking agent (final concentration 1.8%) for 15 min. The reaction was stopped by adding glycine (final concentration 225 mM). The homogenate was cleared by passing through 100-µm nylon cell strainer (BD Falcon) and pelleted by centrifugation at 4000 g, 4°C for 5 min. After twice washing in buffer A1 at 4°C, the pellet was treated with 0.5 ml of complete lysis buffer (15 mM HEPES, pH 7.6; 140 mM NaCl, 1mM EDTA, 0.5 mM EGTA, 1%Triton X-100, 0.5 mM DTT, 0.1% sodium deoxycholate, 0.1% SDS, 0.5 % N-lauroylsarcosine, Roche cOmplete protease inhibitor) and sonicated to break chromatin into fragments with an average length of 200-600 bp. The material was pelleted by centrifugation at 18 000 g for 5 min, and the supernatant fluid was transferred to a new tube. The pellet was treated with the second 0.5-ml portion of lysis buffer, and the preparation was centrifuged at 18 000 g for 5 min. The two portions of the supernatant fluid were pooled, cleared by centrifuging twice at 18 000 g for 10 min, and the resultant chromatin extract (1 ml) was used for ChIP experiments. 9 ml of ChIP dilution buffer (15 mM HEPES, pH 7.6; 140 mM NaCl, 1mM EDTA, 0.5 mM EGTA, 1%Triton X-100, 0.5 mM DTT, Roche cOmplete protease inhibitor), was added to the sheared chromatin together with 50 ul of prewashed Protein A Sepharose beads (GE Healthcare). The samples were incubated at 4 ^{o}C for 1 hour on a rotating platform. The samples were spun at 10,000g to remove the beads and the supernatant was transferred to a fresh tube.

One aliquot (1/10 volume) of chromatin extract after preincubation with Sepharose was kept as a control sample (Input). The appropriate dilution of antibody (1:100 for CrolN, CrolC, Ph, Cg, E(z), GAF and 1:250 for H3K27me3) and 20 µl per sample of Protein A Sepharose (pre-blocked with BSA) were added, then the samples were incubated rocking overnight at 4°C. As a control incubation was made with IgG of nonimmunized rabbit. The next day the beads were washed for 5 minutes on a rocking platform at 4 ^{o}C with wash buffer (20 mM Tris-HCl pH 8, 2 mM EDTA, 150 mM NaCl, 1% Triton X-100, 0.1% SDS), 5 minutes with final wash buffer (20 mM Tris-HCl pH 8, 2 mM EDTA, 500 mM NaCl, 1% Triton X-100, 0.1% SDS). The chromatin was eluted with 500 ul elution buffer (0.1 M $NaHCO_3$, 1% SDS) for 15 minutes at room temperature on a rocking platform. At this point the input sample was defrosted and 500ul of elution buffer was added. The crosslinks were reversed by a 6-hour incubation at 65°C in the presence of 2 µl Recombinant Proteinase K Solution (20 mg/mL, Thermo Fisher Scientific). The DNA was purified using phenol/chloroform followed by ethanol precipitation with 50 µl NaOAc pH5.2, 1,5 ml 100% Ethanol and 4 µl glycogen (20 mg/ml, Sigma). After incubating overnight at -20°C samples were centrifuged at 20,000g for 20 minutes. Pellets were washed with 70% ethanol, air dried and resuspended in nuclease-free water.

Reference: Erokhin M, Gorbenko F, Lomaev D, Mazina MY, Mikhailova A, Garaev AK, Parshikov A, Vorobyeva NE, Georgiev P, Schedl P, Chetverina D. Boundaries potentiate polycomb response element-mediated silencing. BMC Biol. 2021 Jun 2;19(1):113. doi: 10.1186/s12915-021-01047-8. PMID: 34078365; PMCID: PMC8170967.

IV. **ChIP-*seq from larval brains and imaginal discs***

Dissection and fixation of larval brains and discs

Brains and imaginal discs were dissected from 3rd instar larvae (10 larvae per sample) in PBS and placed in Schneider's medium (Invitrogen) on ice in homogenization tubes. The brains and discs were spun at 4000g for 1 minute and the media replaced with 1ml ice cold fixative (2% formaldehyde (Ted Pella Inc, Redding, CA), 50 mM HEPES pH 7.6, 100 mM NaCl, 0.1 mM EDTA, 0.5 mM EGTA supplemented with Roche cOmplete protease inhibitor, EDTA free) Samples were incubated on a rocking platform at room temperature for 15 minutes. The brains and discs were centrifuged at 4000g for 1minute. Fixative was removed and replaced with 1ml stop solution (PBS, 0.01% Triton X-100, 0.125 M glycine supplemented with Roche cOmplete protease inhibitor), and incubated rocking 10 minutes at room temperature, followed by centrifugation as above, then 2×10-minute washes with a post fix wash solution (50mM Tris, 10 mM EDTA, 0.5 mM EGTA, 0.25% Triton X-100 supplemented with Roche cOmplete protease inhibitor). The

brains and discs were centrifuged at 4000g for 1 minutes then stored at −80°C in 50μl storage solution (10 mM Tris-HCl pH 8.0, 1 mM EDTA, 0.5 mM EGTA).

ChIP-seq

Brain and disc samples were thawed on ice then centrifuged at 4000g for 1 minute. The storage buffer was replaced with 300μl nuclear lysis/sonication buffer (50mM Tris/HCl pH8.0, 50mM NaCl,1mM EDTA 0.1%SDS, 1% Triton X-100, 0.1% deoxycholate, Roche cOmplete protease inhibitor). The samples were homogenized with disposable pestles then transferred into 500μl tubes and sonicated using a (QSonica) Q800R3 sonicator at 70% amplitude for 30secs on, 30secs off for a total on time of 22 minutes giving an average size range of 200-500bp. Samples were centrifuged at 20,000g for 10 minutes and the supernatant was transferred to a new tube. A percentage of the sample was removed as the input control and stored at -20°C. 700μl of ChIP dilution buffer (16.7mM Tris-HCl pH8, 1.2mM EDTA, 167mM NaCl, 1.1% Triton X-100, 0.01% SDS, Roche cOmplete protease inhibitor), was added to the sheared chromatin together with 80μl of prewashed Protein A Sepharose beads (GE Healthcare). The samples were incubated at 4 °C for 1 hour on a rotating platform. The samples were spun at 10,000g to remove the beads and the supernatant was transferred to a fresh tube. The appropriate dilution of antibody was added (1:100 CrolN, 1:100 CrolC, 1:100 E(z), 1:100 Ph, 1:100 H3K27me3, 1:100 Cg), then the samples were incubated rocking overnight at 4°C. The next day 60μl of prewashed Protein A Sepharose beads (GE Healthcare) were added to each sample and incubated 1 hour on a rocking platform at 4°C. The beads were washed for 5 minutes on a rocking platform at 4 °C with low salt wash buffer (20 mM Tris-HCl pH 8, 2 mM EDTA, 150 mM NaCl, 1% Triton X-100, 0.1% SDS), 5 minutes with high salt wash buffer (20 mM Tris-HCl pH 8, 2 mM EDTA, 500 mM NaCl, 1% Triton X-100, 0.1% SDS), 5 minutes with LiCl wash buffer (10 mMTris-HCl pH 8, 1 mM EDTA, 250 mM LiCl, 1% NP-40, 1% deoxycholate), 2 X 5 minutes washes at room temperature with 1 X TE buffer pH 8. The chromatin was eluted twice with 250μl elution buffer (0.1 M$NaHCO_3$, 1% SDS) for 15 minutes at room temperature on a rocking platform. At this point the input sample was defrosted and 500μl of elution buffer was added. The crosslinks were reversed by the addition of 20μl of 5MNaCl to all the samples followed by a 4 hour incubation at 65°C. 10μl of 0.5M EDTA and 20μl of 1M tris-HCl pH 6.5 was added to each sample together with 2μl Roche PCR grade 18.5mg/ml Proteinase K. The samples were incubated for 1 hour at 50°C. The DNA was purified using phenol/chloroform followed by ethanol precipitation with 50 μl NaOAc pH5.2, 1ml 100% Ethanol and 2 μl pellet paint co-precipitant (Millipore). After incubating overnight at -20°C samples were centrifuged at 20,000g for 20 minutes. Pellets were washed with 70% ethanol, air dried and resuspended in 200 μl of nuclease-free water.

DNA concentration of 5ul was measured using a Qubit 3.0 fluorometer (Invitrogen). 1.5ng of starting material/sample was used to make each library. ChIP-seq libraries were obtained using the NEBNext Ultra™ II DNA library preparation kit (New England Biolabs) or with the Thruplex DNA-seq and single index kits (Takara) following the manufacturer's instructions. Samples were sequenced by 50bp or 100bp single-end sequencing with HiSeq2500 (Illumina) or with NovaSeq6000 sequencer.

V. Sequences of DNA-fragments obtained by PCR for EMSA

The DNA plasmids with 20xG and Control insertions were made on the basis of pBluescript SK + vector. For the PCR the M13rev and M13dir primers containing the Cy5 fluorophore were used. The DNA fragments obtained by PCR are shown.

20xG

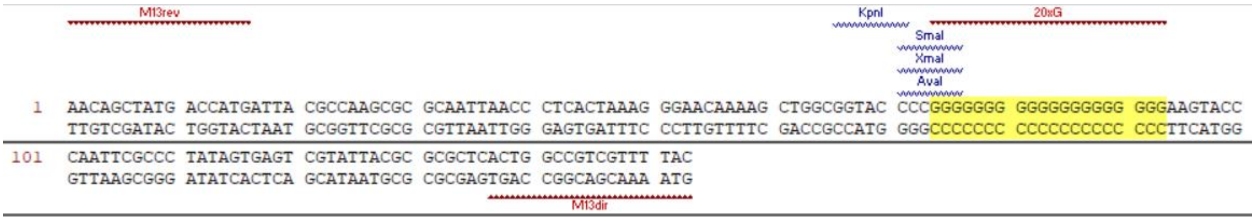

Control

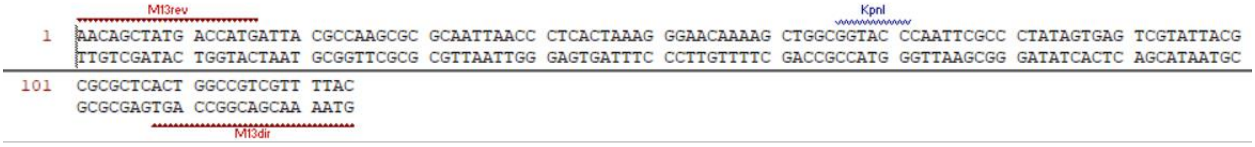

20bp-evePRE

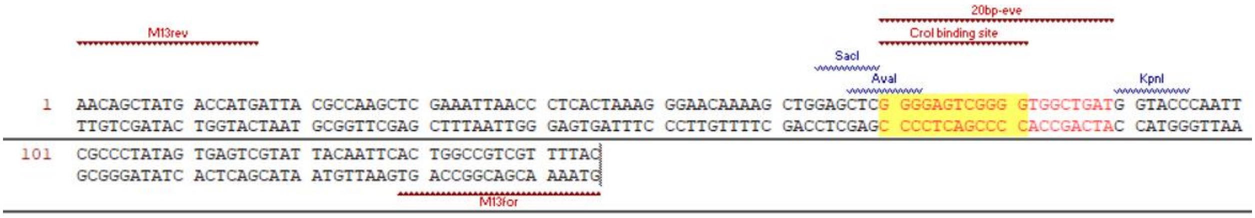

20bp- evePREmut

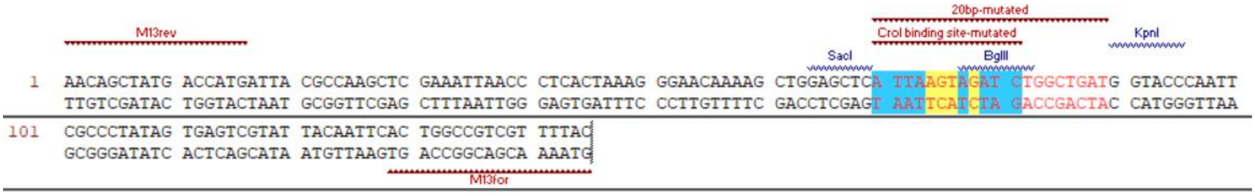

Supplement: gkad336_Supplemental_Files [file gkad336_supplemental_files.zip › Erokhin et al - Supplementary File 4. Supplementary Material and Methods.pdf]
