# Supplementary material for: Crol contributes to PRE-mediated repression and Polycomb group proteins recruitment in Drosophila
Source: Nucleic Acids Res. 2023 May 4;51(12):6087–100. doi: 10.1093/nar/gkad336 (PMC10325914; doi:10.1093/nar/gkad336)
Supplement: gkad336_Supplemental_Files [file gkad336_supplemental_files.zip › Erokhin et al - Supplementary File 5. Primers used in study.pdf]

Supplementary file 5

Erokhin et al.

Crol contributes to PRE-mediated repression and Polycomb group proteins recruitment in *Drosophila*

Table S1 Sequences of primers used for ChIP-qPCR for detection of the Crol binding to the genome PREs

Primer Name	Primer sequence 5′ to 3′
*bxd*PRE dir	aagagcaaggcgaaagagagc
*bxd*PRE rev	cgttttaagtgcgactgagatgg
*bx*PRE dir	gcgtccagtgaaaatcggaatga
*bx*PRE rev	caaaacaggcgagcatataagcg
*Fab7*PRE dir	ggaataccgcactgtcgtagg
*Fab7*PRE rev	gcagccatcatggatgtgaa
*en*PRE2 dir	agatggcatgtggctctccc
*en*PRE2 rev	aactgtgtccccagcgaactg
*eve*PRE dir	agatggcatgtggctctctccc
*eve*PRE rev	tttgggtctgaggcggttcc
Ras64B dir	gagggattcctgctcgtcttcg
Ras64B rev	gtcgcacttgttacccaccatc
Tub dir	agttcaccgctatgttca
Tub rev	cgcaaaacattgatcgag

Table S2 Sequences of primers used for ChIP-qPCR from transgenic constructs

Primer Name	Primer sequence 5′ to 3′
1dir	gtcattctgagaatagtgtatgcg
1rev	ggtaagatccttgagagttttcg
2dir	gtaaaacgacggccagt
2rev	gttctgcgtttctatggagttt
3dir	tccaccaaactctgctgtacag
3rev	tagaattaattcgagctcgccc
4dir	gcaaatgtcagcacacgatcat
4rev	gtgggctcatcgcagatca
