# Supplementary material for: Crol contributes to PRE-mediated repression and Polycomb group proteins recruitment in Drosophila
Source: Nucleic Acids Res. 2023 May 4;51(12):6087–100. doi: 10.1093/nar/gkad336 (PMC10325914; doi:10.1093/nar/gkad336)
Supplement: gkad336_Supplemental_Files [file gkad336_supplemental_files.zip › Erokhin et al - Supplementary File 6. Supplementary Figures.pdf]

Supplementary file 6

Erokhin et al.

Crol contributes to PRE-mediated repression and Polycomb group proteins recruitment in *Drosophila*

This file includes Supplementary Figures S1- S12.

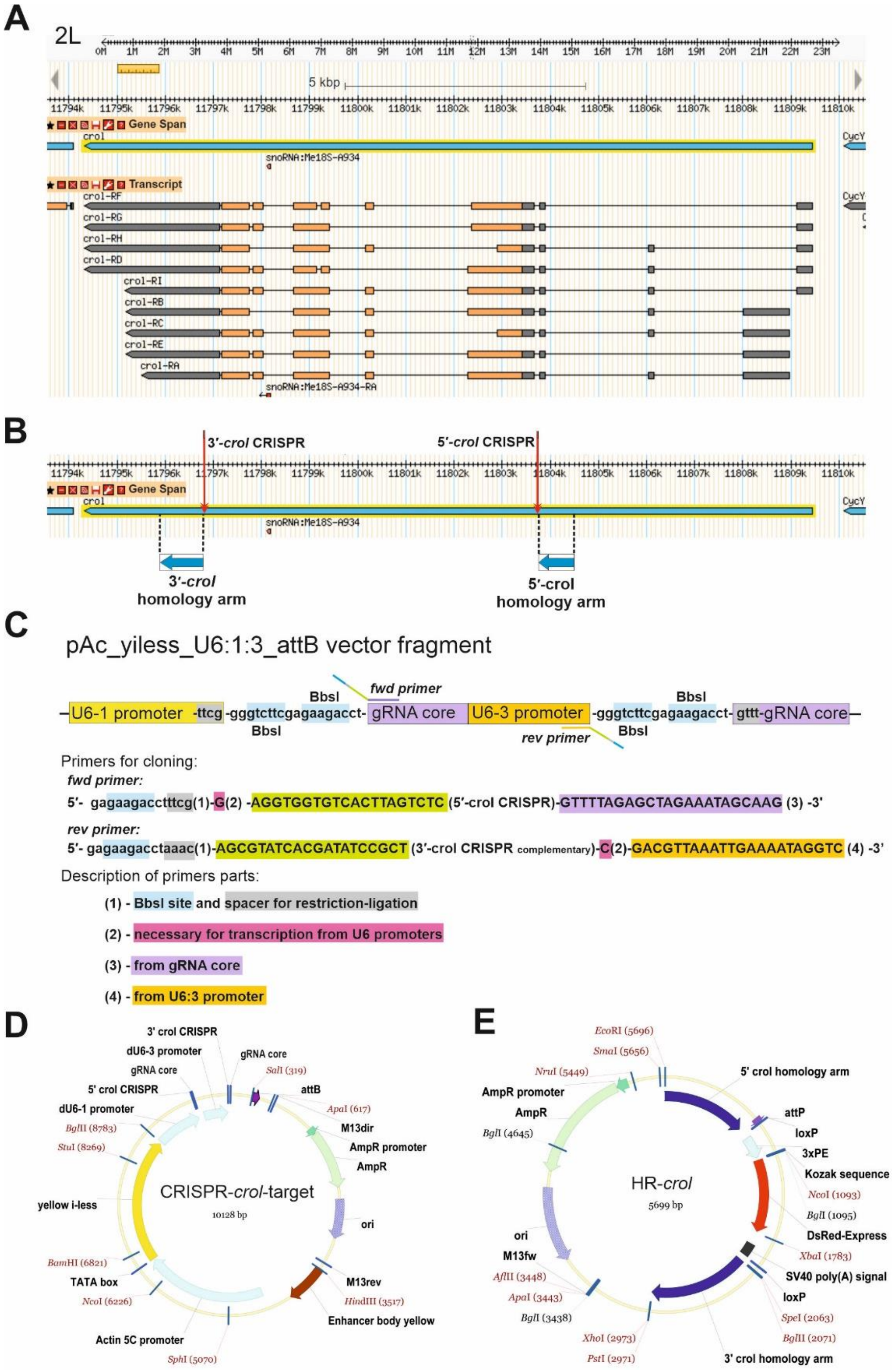

Supplementary Figure S1. The schemes of *crol* gene regions and plasmids used for knockout (KO) of the *crol* gene by CRISPR/Cas9 technology. **A**) The *crol* genome region and transcribed *crol* isoforms. **B**) 3′ and 5′ *crol* homology arms used for homologous recombination and 3′ and 5′ *crol* CRISPRs positions are indicated. **C**) pAc-y-iless vector fragment used for cloning of the 5′ and 3′ *crol* CRISPRs is shown. Full length primers sequence and their detailed structure are indicated. **D**) The scheme of CRISPR-*crol*-target plasmid with 3′ and 5′ CRISPRs is shown. This plasmid is made based on pAc-y-iless vector. **E**) The scheme of HR-*crol* plasmid with *crol*-homology arms and DsRed gene for replacement is shown.

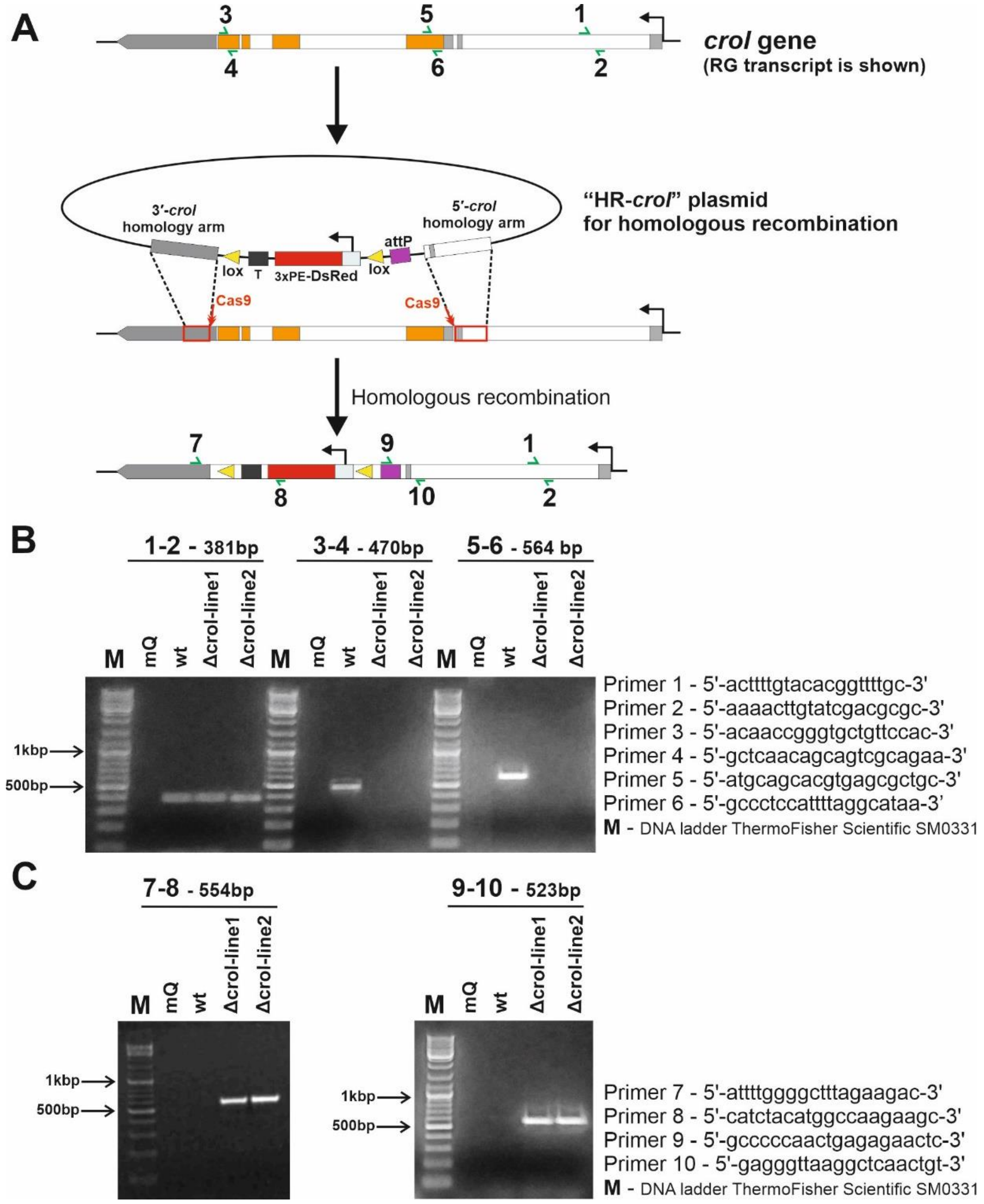

Supplementary Figure S2. Generation and validation of fly lines with knockout (KO) of the *crol* gene. **A**) Scheme of experiment with homologous recombination of the *crol* gene after co-injection of CRISPR-*crol*-target and HR-*crol* plasmids into Cas9-expressing flies. Color designations: orange - Crol coding part; grey – UTRs; white – introns; red – DsRed; black – SV40-poly(A) signal ("T"); purple – attP; yellow – lox-sites. attP/attB and Cre/lox recombination were not used in current study. The numbers (1 -10) indicate the positions of primers used for the analysis. **B**) Confirmation of the *crol* gene knockout (*crol* KO) in $crol^-/crol^-$ homozygous flies with PCR. y^-w^{1118} flies were used as the *wild type (wt)* control. Primer pairs 3-4 and 5-6 are from *crol* coding part that is deleted in *crol* KO flies. Primer pair 1-2 is from remaining in *crol* KO flies (*crol* first intron fragment) was used as a PCR genomic DNA quality control. **C**) Confirmation of the correct homologous recombination using PCR with construct specific primers (Primer pair 7-8 – from DsRed and 3′-*crol* UTR; Primer pair 9-10 – from attB and *crol* intron).

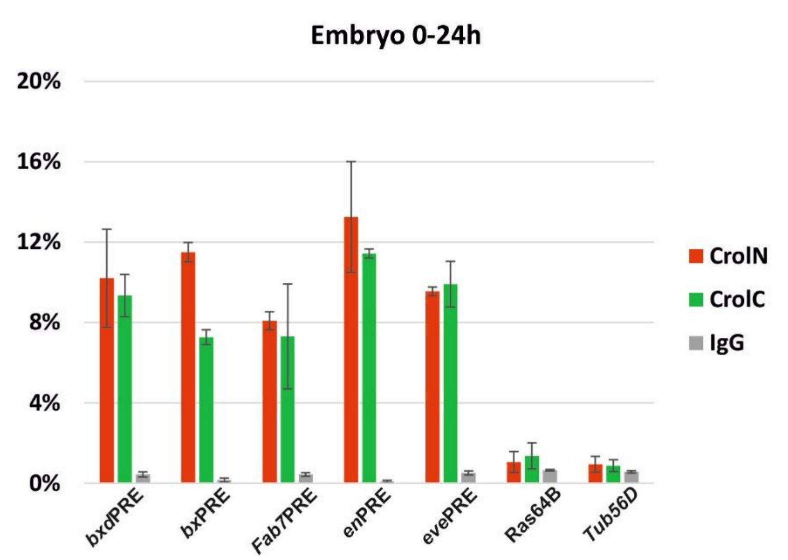

Supplementary Figure S3. Crol enrichment at well-characterized PREs during embryogenesis. Diagrams summarize the results of X-ChIP with Crol-N or Crol-C rabbit antibody, or with IgG from a non-immunized rabbit as a negative control. The X-ChIP experiments were performed with chromatin isolated from the 0-24 h wild-type Oregon embryos. The ordinate shows the percentage of target sequences in the immunoprecipitated material relative to the input DNA. X-ChIP was analyzed by real-time qPCR with primers specific to the PREs: *bxd*PRE, *bx*PRE, *Fab7*PRE, *en*PRE, *eve*PRE, or to negative genome controls: the coding part of Ras64B and tubulin genes (indicated on the abscissa).

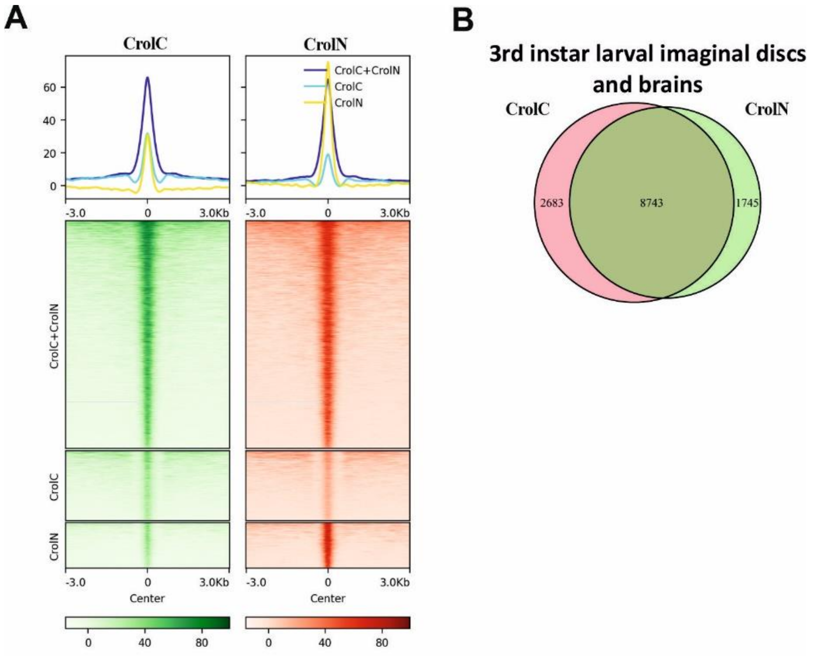

Supplementary Figure S4. A) Heatmaps and **B**) Venn diagram showing the overlap between Crol peaks obtained by ChIP-seq with CrolN or CrolC antibodies using chromatin isolated from wild-type 3rd instar larval brains and imaginal discs.

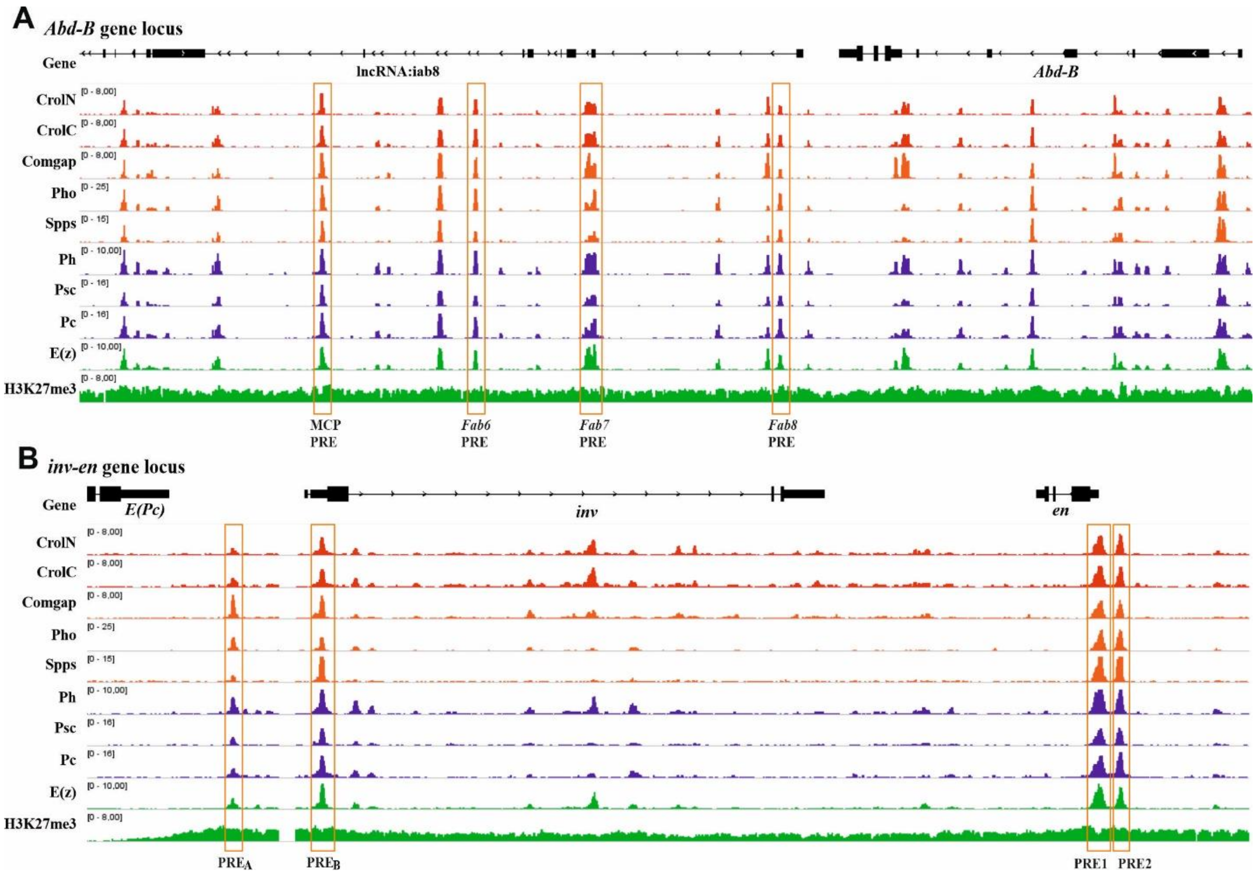

Supplementary Figure S5. ChIP-seq profiles visualized as IGV tracks for Crol (CrolN and CrolC antibodies), Combgap, Pho, Spps, Ph, Psc, Pc, E(z), and H3K27me3 histone modification. Chromatin was isolated from 3rd instar wild-type larval brains and imaginal discs. **A**) *Abd-B* gene locus. Characterized *MCP*PRE, *Fab6*PRE, *Fab7*PRE, and *Fab8*PRE are highlighted by orange boxes. **B**) *inv-en* gene locus. Characterized PRE_A, PRE_B, PRE1, and PRE2 are highlighted by orange boxes.

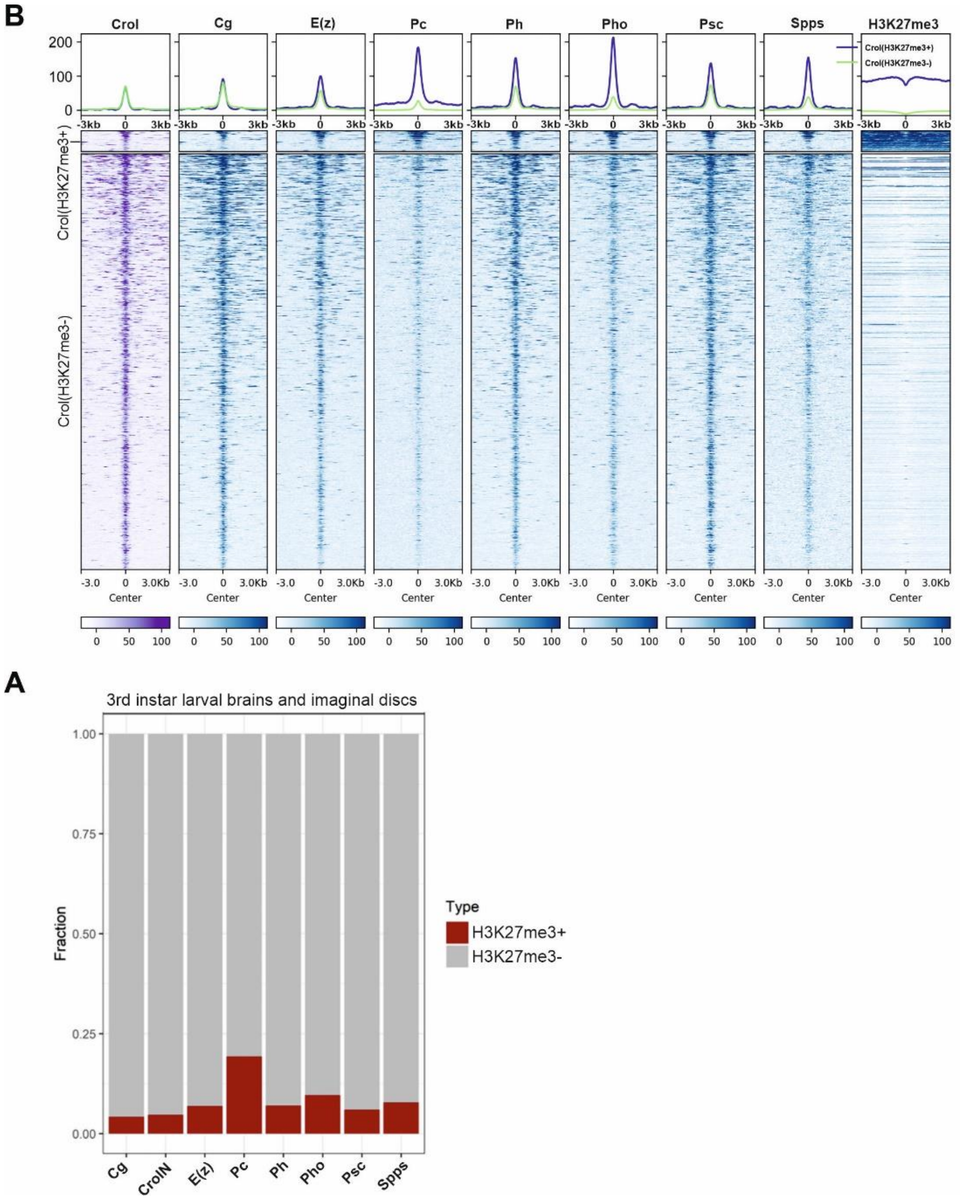

Supplementary Figure S6. PcG proteins are bound within and outside of the H3K27me3 domains. **A**) Heatmaps show the colocalizations of Crol, Cg, E(z), Pc, Ph, Pho, Psc, and Spps within the H3K27me3+ and H3K27me3- domains. Chromatin was isolated from wild-type 3rd instar larval brains and imaginal discs. **B**) Fractions of Cg, Crol, E(z), Pc, Ph, Pho, Psc, and Spps within and without the chromatin marked by H3K27me3 histone modification.

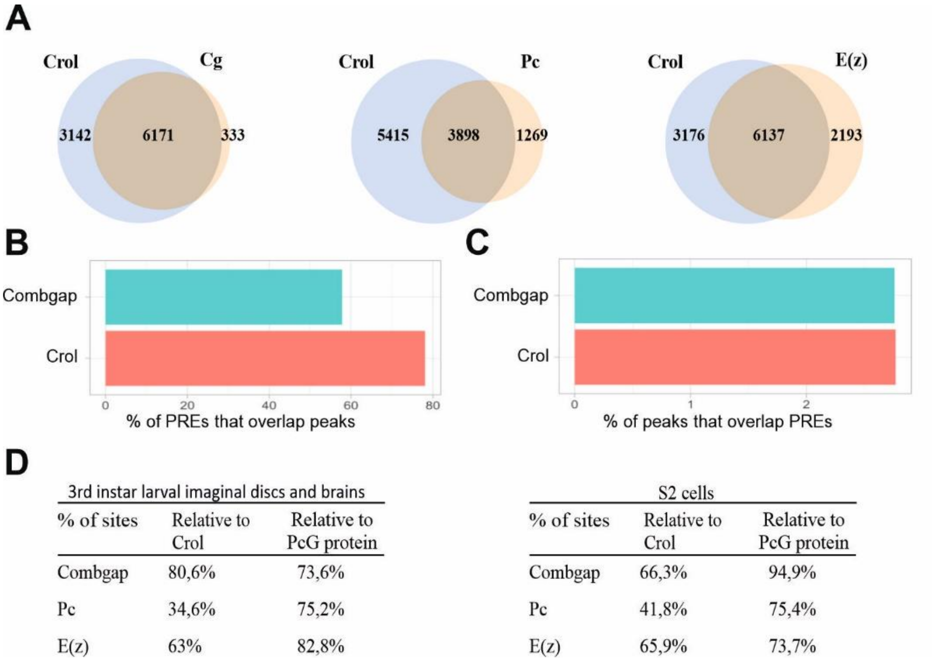

Supplementary Figure S7. Crol colocalizes with PcG proteins and PREs genome-wide in S2 Drosophila cells. **A**) Venn diagrams showing the overlap between Crol and Combgap, Pc, and E(z). **B**) Percent of PREs with Crol and Combgap peaks. PREs were defined as regions with simultaneous binding of E(z), Pc, and the presence of H3K27me3 histone modification. **C**) Percent of Crol and Combgap peaks within the PREs. **D**) Comparison of Crol overlap with PcG proteins in 3rd instar larval brains and imaginal discs (left) and S2 cells (right).

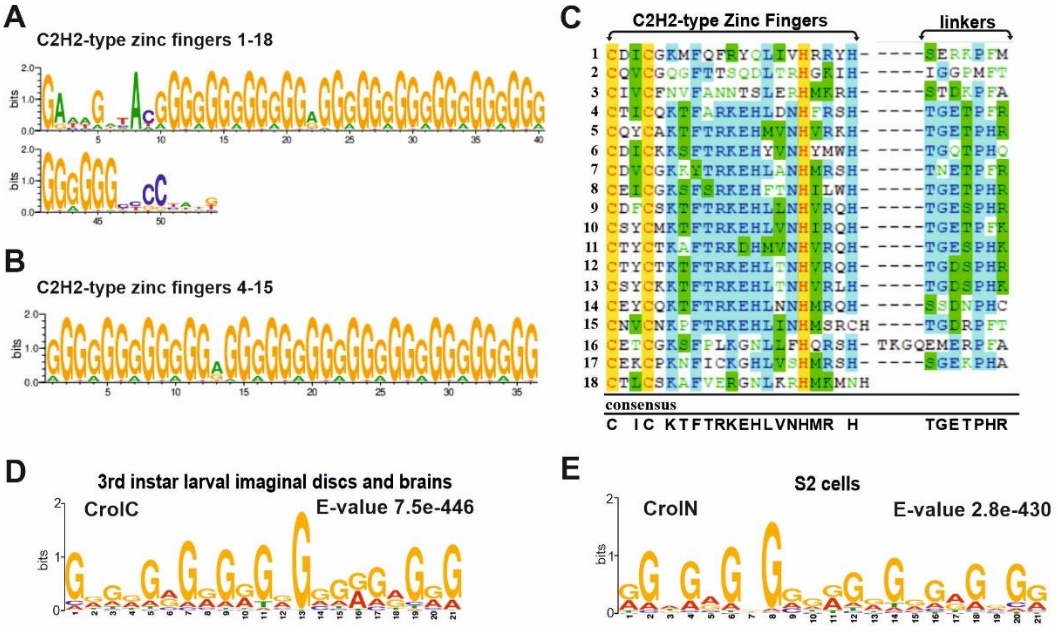

Supplementary Figure S8. Crol is C2H2-type zinc finger protein that binds poly(G)-rich sequences. **A, B**) Crol predicted DNA-binding motif using bioinformatic modeling at zf.Princeton.edu using Zinc finger motifs 1-18 (A) and Zinc finger motifs 4-15 (B). The poly(G) motif is predicted for 4-15 Crol zinc fingers, while flanking zinc fingers do not display such specificity. **C**) Alignment of Crol zinc fingers and linkers between them. Designations: The identical amino acids are highlighted in yellow; conservative – in blue; block of similar – in green; while weakly similar have green foreground color. Zinc fingers 4-15 have the highest homology and contain the same amino acids in positions 10, 12, 13, 16 (positions that are significant for

DNA binding specificity). In addition, the linkers between zinc fingers 4-15 are of higher homology than flanking ones. This assumes that zinc fingers 4-15 might be responsible for the predicted specificity for poly(G)-binding. **D**) Crol estimated DNA-binding motif using ChIP-seq obtained on 3rd instar larval brains and imaginal discs with CrolC antibodies. **E**) Crol estimated DNA-binding motif using ChIP-seq obtained on S2 Drosophila cells with CrolN antibodies.

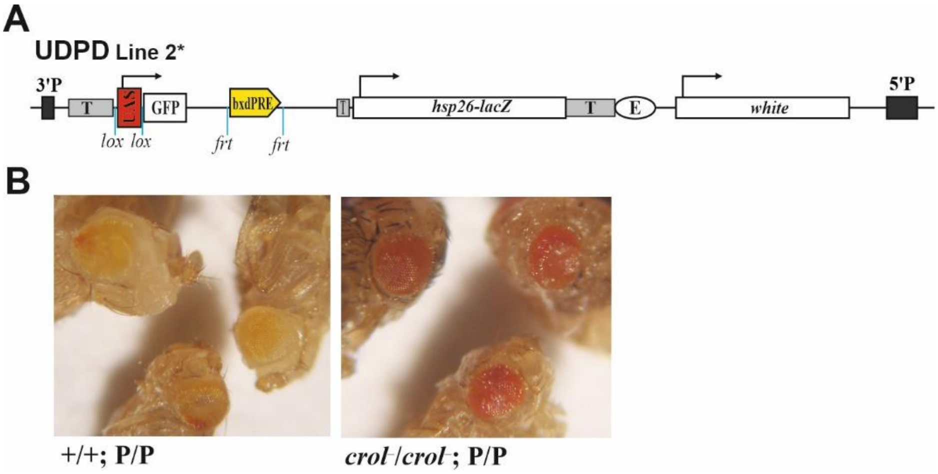

Supplementary Figure S9. *crol*-KO suppress the *bxd*PRE-depending silencing in transgenes. **A**) The scheme of the UDPD transgene construct containing *bxd*PRE. This transgene contains an upstream activating sequence element linked to the *hsp70* promoter (UAS). The 656-bp *bxd*PRE is between the UAS and the *hsp26-lacZ* and *white* reporter genes. Other labels: "T" - terminators of transcription; "E" - enhancer of the *white* gene; 3'P and 5'P - 3'- and 5'-ends of P-element containing inverted repeats required for transposase dependent integration of the transgenes into the genome. The UDPD transgene and corresponding transgenic lines were previously described in Erokhin et al., PNAS, 2015. In current study we used UDPD transgenic line#2 inserted on the third chromosome. **B**) The eye phenotypes of UDPD homozygous control flies (+/+; P/P, left) or UDPD flies with *crol* KO background ($crol^-/crol^-$; P/P, right) at the pharate adult stage are shown. The phenotype of the eyes of the UDPD transgenic flies is darker upon *crol* KO indicating the release of silencing.

Erokhin M, Elizar'ev P, Parshikov A, Schedl P, Georgiev P, Chetverina D. Transcriptional read-through is not sufficient to induce an epigenetic switch in the silencing activity of Polycomb response elements. Proc Natl Acad Sci U S A. 2015 Dec 1;112(48):14930-5. doi: 10.1073/pnas.1515276112. Epub 2015 Oct 26. PMID: 26504232; PMCID: PMC4672805.

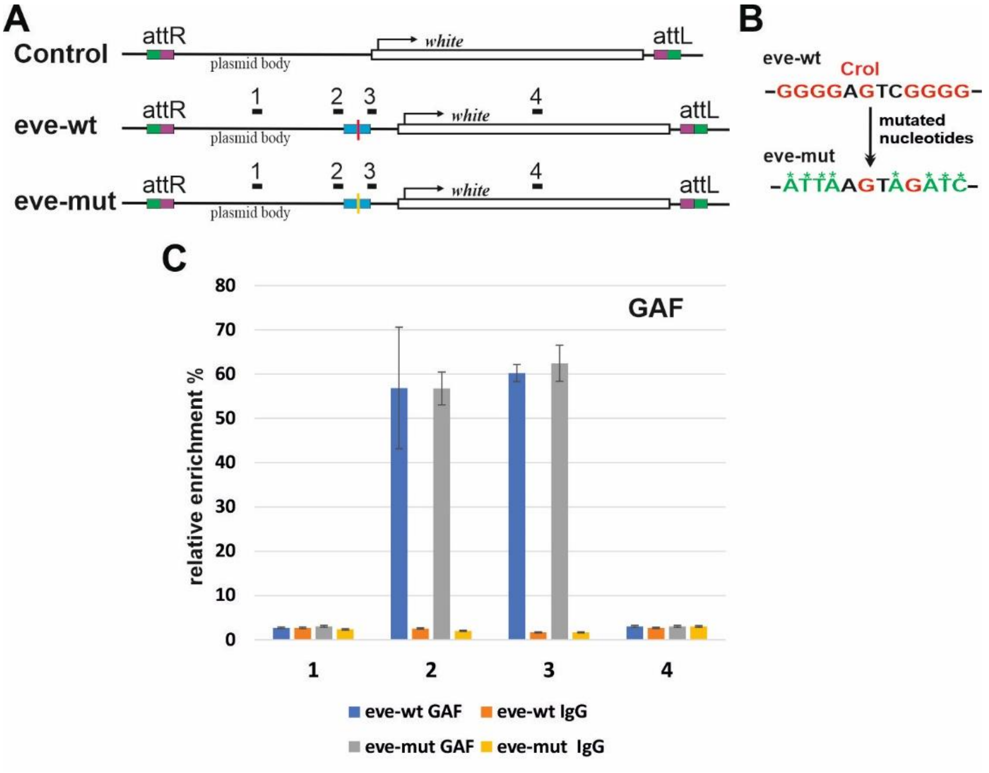

Supplementary Figure S10. Mutation of CroI binding site in evePRE containing transgene doesn't affect the binding of GAF protein. **A**) The scheme of transgene constructs. **Control** - *white* reporter gene and attB site required for transgene integration into the attP insertion site. **eve-wt** – wild-type 344bp *eve*PRE, **eve-mut** –344bp *eve*PRE with mutated CroI-binding sites. Other elements in **eve-wt** and **eve-mut** the same as in **Control** construct. Numbers on top of the construct schemes (1, 2, 3, and 4) indicate regions amplified by qPCR in X-ChIP experiments. The transgenes were integrated into attP2 insertion point. **B**) The nucleotides mutated in 344bp **eve-mut** were the same as in 20bp-mut tested in EMSA in Figure 3J. **C**) The ChIP experiments were performed with chromatin isolated from 3rd instar wild-type larval homozygous for the **eve-wt** or **eve-mut** transgenes. The X-ChIPs were performed with anti-GAF antibody or with IgG. The ordinate shows the percentage of target sequences in the immunoprecipitated material relative to the input DNA and normalized to the positive control—a sequence adjacent to the endogenous *bxd*PRE in BX-C (*bxd*PRE-Genome). The transgene specific regions (1, 2, 3, and 4) are indicated on the abscissa. Vertical lines indicate SDs.

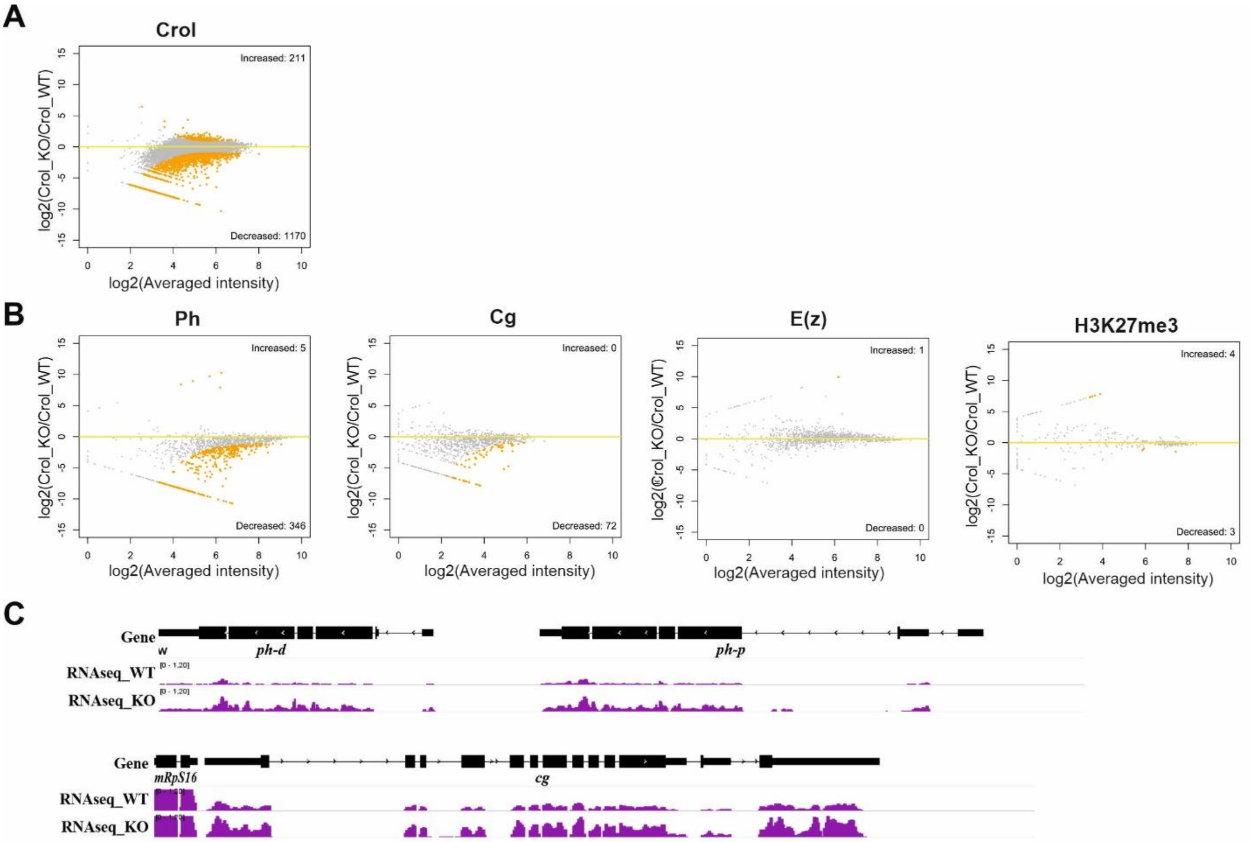

Supplementary Fig. S11. Crol KO affects the Ph and Cg binding genome-wide. **A**) The MA-plots show the change in the binding of the Crol in *crol* KO vs wild-type 3rd instar larval brains and imaginal discs. Peaks showing significant differential binding (FDR<0.05) as identified by DiffBind are marked in orange**.** **B**) The MA-plots show the changes in the binding of the Ph, Cg, E(z), and H3K27me3 on the 1170 Crol decreasing peaks in *crol* KO vs wild-type 3rd instar larval brains and imaginal discs. Peaks showing significant differential binding (FDR<0.05) as identified by DiffBind are marked in orange**. C**) RNAseq profiles of the the wild-type (WT) and *crol* KO third instar whole larvae are shown for *ph-p/ph-d* (up) and *cg* (down) genes.

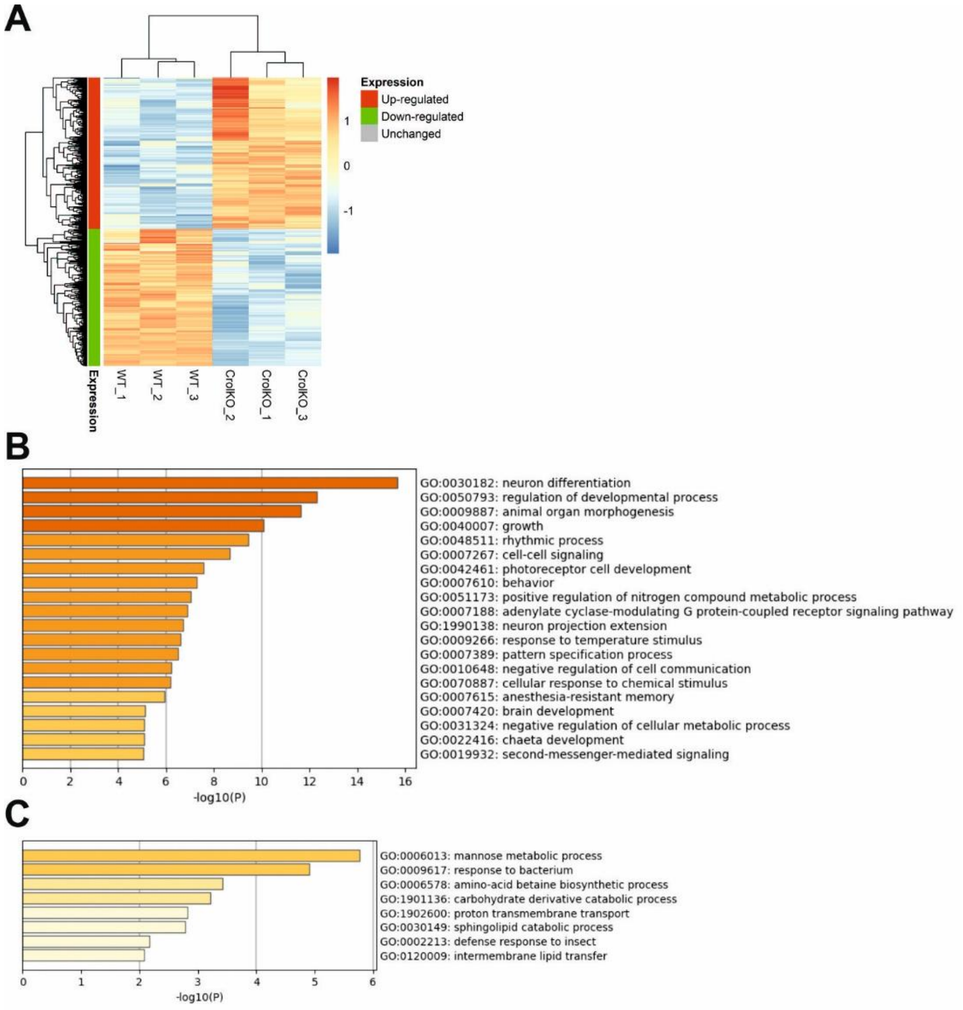

Supplementary Figure S12. RNA-seq analysis of the wild-type and *crol* KO flies. **A)** Heatmap for the expression patterns of the DEGs between *crol* KO vs wild-type (WT) third instar larvae. The gradient color indicates the row z-score. **B)** Gene Ontology analysis of genes up-regulated upon *crol* KO. **C)** Gene Ontology analysis of genes down-regulated upon *crol* KO.
